# Tailoring Composite Microstructure Through Milling for Dry‐Processed Sulfide‐Based Solid‐State Battery Cathodes

**DOI:** 10.1002/smll.202507279

**Published:** 2025-09-05

**Authors:** Finn Frankenberg, Carina A. Heck, Maximilian Kissel, Martin A. Lange, Vasiliki Faka, Alexander Diener, Philipp Haase, Peter Michalowski, Wolfgang G. Zeier, Jürgen Janek, Arno Kwade

**Affiliations:** ^1^ Technische Universität Braunschweig Institute for Particle Technology Volkmaroder Straße 5 38104 Braunschweig Lower Saxony Germany; ^2^ Justus‐Liebig‐Universität Gießen Institute of Physical Chemistry & Center for Materials Research Heinrich‐Buff‐Ring 17, Hesse 35392 Gießen Germany; ^3^ Universität Münster Institute of Inorganic and Analytical Chemistry Corrensstraße 28‐30 48149 Münster North Rhine‐Westphalia Germany; ^4^ Forschungszentrum Jülich GmbH Institute of Energy Materials and Devices (IMD) IMD 4: Helmholtz‐Institut Münster Ionics in Energy Storage 48149 Münster North Rhine‐Westphalia Germany

**Keywords:** chemical process engineering, DEM simulation, microstructure, process–structure–property, solid‐state battery

## Abstract

While the effects of new solid electrolytes and active materials in cathode composites for solid‐state batteries are being intensively researched, little is known about the influence of mechanical processing on the properties of these composites. Here, the influence of mechanical process parameters on the production of Li_6_PS_5_Cl and LiNi_0.83_Co_0.11_Mn_0.06_O_2_ composite cathodes applying a planetary ball milling process is systematically investigated. It is shown that the milling process has a significant influence on the microstructure of the composite by affecting the solid electrolyte particle size and the formation of electrolyte‐active material aggregates. The combination of experimental results with discrete element simulations shows that changes in microstructure with increasing energy input result in an increase in the density of heterocontacts, which improves the electrochemical performance. However, if the energy input is too high, a decrease in the crystallite size of Li_6_PS_5_Cl and an increase in strain in LiNi_0.83_Co_0.11_Mn_0.06_O_2_ have a negative impact on the electrochemical performance. Subsequent dry film production of the pre‐milled composites reveals that a non‐optimized composite can be partially compensated by the high shear stresses acting during dry film production. Overall, the paramount importance of precisely controlling the milling process for the production of cathode composites is demonstrated.

## Graphical abstract

This study shows that milling of LiNi_0.83_Co_0.11_Mn_0.06_O_2_‐Li_6_PS_5_Cl composites strongly influences the microstructure and electrochemical performance. Key factors include stress energy and specific energy input. Optimized milling improves aggregate formation, porosity, and particle contact, thereby enhancing performance, whereas excessive specific energy input leads to degradation. Process engineering in combination with material science is essential for scalable, high‐performance solid‐state battery film production.

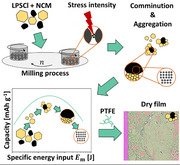

## 1 Introduction

Solid‐state batteries (SSBs) are emerging as a safer and more efficient alternative to conventional lithium‐ion batteries (LIBs), offering higher energy and power densities.^[^ ^1^, ^2^ ^]^ Achieving these advancements requires new material systems, solid electrolytes with superior ionic conductivities, and the successful implementation of the lithium anode.^[^ ^3^, ^4^, ^5^ ^]^ However, also scalable production processes with which SSBs will be produced on a large scale in industry must be considered.^[^ ^6^, ^7^ ^]^ In this context, machines such as mixers,^[^ ^8^ ^]^ extruders,^[^ ^7^ ^]^ and calenders^[^ ^9^ ^]^ impact the microstructure of the battery electrodes, which in turn affects electrochemical performance.^[^ ^10^, ^11^, ^12^, ^13^, ^14^, ^15^ ^]^

In the case of SSBs, mixing plays an especially critical role. Unlike conventional electrodes, where the active material is typically mixed with binder and carbon black,^[^ ^11^ ^]^ the solid electrolyte is incorporated directly into the SSB composite. As mixing in the case of SSBs often comes along with structural changes of the components, it is here referred to as *milling*. Proper milling defines the distribution of active material and solid electrolyte, impacting the microstructure and performance of the final electrode.^[^ ^16^ ^]^ Of utmost importance on the cathode side is the mechanical contact between the solid electrolyte and active material particles (*heterocontacts*) as well as contacts within each particle species, as it is key to ensuring effective lithium ion and electron transport.^[^ ^2^, ^17^ ^]^

The mechanical contact can be achieved particularly well with soft sulfide‐based solid electrolytes, which have emerged as a promising candidate for SSBs due to their combination of good processability and high ionic conductivities (1–10 mS cm^−1^).^[^ ^3^, ^18^, ^19^, ^20^ ^]^ Smaller particles of these electrolytes have proven to enhance effective ionic conductivity in the cathode composite by improving particle packing, reducing tortuosity, and improving the overall electrochemical performance.^[^ ^21^, ^22^ ^]^ Furthermore, they provide better crack suppression and a smoother interface at the electrolyte/electrode and lithium metal interfaces, which helps improve stability of the SSB.^[^ ^23^, ^24^ ^]^ However, after synthesizing sulfide electrolytes or when purchasing them commercially, the particles are often large and unevenly distributed, which makes comminution necessary.^[^ ^9^, ^25^ ^]^ Initial studies on the wet comminution of the sulfide electrolyte Li_5.5_PS_4.5_Cl_1.5_ in a planetary ball mill show that particle size reduction of the electrolyte improves effective ionic conductivity in the composite.^[^ ^7^, ^26^ ^]^ However, it decreases bulk ionic conductivity, presenting a trade‐off that requires further optimization. A similar trend was observed for dry pre‐comminution of Li_5.5_PS_4.5_Cl_1.5_ for which a small frequency ball mill was compared with a planetary ball mill.^[^ ^27^ ^]^ Process parameters such as rotational speed and milling time significantly impacted the solid electrolyte microstructure and, consequently, the cathode composite. Discrete element method (DEM) simulations modeled both processes, calculating the specific energy input *E*_m_ and correlating it with the coherence length, revealing increasing disorder of the electrolyte with higher energy input.

Previous research has primarily focused on the pre‐comminution of the sole electrolyte, leaving the subsequent milling of the cathode composite largely unexplored. To ensure consistency and scalability, these milling processes should be performed using mills rather than a mortar and pestle,^[^ ^27^, ^28^, ^29^, ^30^ ^]^ which is still commonly used in research but leads to significant variations in the quality of the composite.^[^ ^31^ ^]^ Various laboratory mills, such as planetary ball mills or frequency mills, can be used, or later in large‐scale production, stirred media mills. In addition to the choice of mill, mill‐specific parameters such as the milling bead size, the milling time, the rotational speed, and the filling degree result in a large parameter space, each significantly impacting the cathode composite microstructure. In particular, the rotational speed, and thus the acting stress intensity on the particles, strongly changes the solid electrolyte particle size within the composite, thereby shaping its microstructure and electrochemical performance.^[^ ^8^ ^]^

Most studies focus on building powder cells with the cathode composite, while binder‐based films are more relevant for industrial SSB production.^[^ ^9^, ^15^, ^32^ ^]^ Binder addition is typically done either by solvent‐based processing or dry processing. Solvent‐based methods enable simple binder distribution^[^ ^33^, ^34^ ^]^ but may lead to binder coverage of the solid electrolyte and active material particles, potentially isolating them and thereby negatively affecting electrochemical performance.^[^ ^35^, ^36^ ^]^ Dry processing with fibril‐like polytetrafluoroethylene (**PTFE**) overcomes these challenges^[^ ^37^ ^]^ and eliminates solvent‐related environmental concerns, offering benefits such as lower costs and the removal of drying steps.^[^ ^14^, ^38^ ^]^ Although the dry film production process itself has been previously demonstrated,^[^ ^37^ ^]^ the influence of the pre‐milling process on the dry film production process and on the resulting microstructure and therefore electrochemical performance of the SSB has not been investigated yet.

For this reason, the aim of this study is to address key questions that remain open in current research: How do process parameters such as milling time, bead size, and rotational speed influence the direct milling of active cathode materials with solid electrolytes? Can an optimal process window be identified to enhance the electrochemical performance of the cathode composite, and how can simulations assist in pinpointing this window? Furthermore, how do comminution and initial milling affect the production of binder‐based cathode films formed via dry film formation?

**Figure 1:**
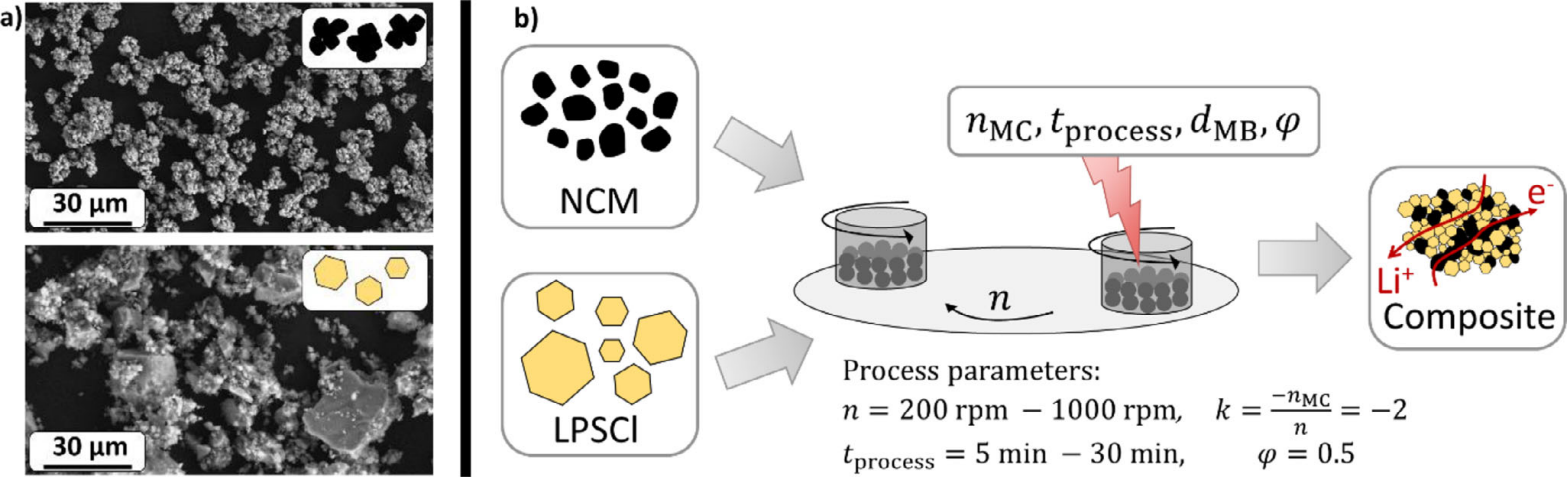
a) SEM images of the starting materials NCM (top) and LPSCl (bottom). b) Schematic representation for the planetary ball milling process of NCM – LPSCl composites showing the influencing factors: rotational speed ***n***, milling time ***t***_**process**_, milling bead size ***d***_**MB**_, and filling ratio ***φ*** on the cathode composite.

Therefore, a systematic investigation on the milling of Li_6_PS_5_Cl (**LPSCl**) and single‐crystalline LiNi_0.83_Co_0.11_Mn_0.06_O_2_ (**NCM**) in a planetary ball mill (**Figure** **1**a,b) was conducted. The composites were prepared with a constant milling bead filling but at different rotational speeds and milling times. The resulting composites were investigated in terms of particle size, resulting microstructure, possible degradation, and electrochemical performance. The ball milling process was simulated by DEM simulations to gain insights into the milling process of cathode composites and to calculate mill‐specific quantities. Furthermore, the pre‐milled cathode composites were used for dry film production of cathode films using PTFE as a binder to quantify the effect of pre‐milling on the later cathode film. Our results show that pre‐milling is crucial for improved performance and that process engineering is key to SSB production. As processing significantly alters the microstructural properties of materials, it must be treated as a central element of material design.

## 2 Results and Discussion

### 2.1 Brief Introduction to the Theory of Milling in (Planetary) Ball Mills

The stress on the cathode composite of NCM and LPSCl in a planetary ball mill varies significantly due to the change in rotational speed and milling time. Both process parameters ensure that the microstructure of the later composite cathode is modified. The influence of process parameters of mills can be described using the model parameters, mean stress energy $\bar{SE}$ and stress frequency *SF*.^[^ ^39^ ^]^ The mean stress energy represents the mean dissipated energy during milling bead collisions or collisions of milling beads with the milling chamber wall.^[^ ^40^, ^41^ ^]^ This collision energy increases with an increase in rotational speed and is a measure of the intensity with which the cathode composite is stressed in the mill. Typically, stress intensity $\bar{SI}$, defined as the dissipated energy per captured active mass between colliding beads *m*_active_ ($SI=\frac{\bar{SE}}{m_{\mathrm{active}}}$),^[^ ^42^ ^]^ is also taken into account. However, since this study assumes a comparable captured mass, the stress energy $\bar{SE}$ is used here as a measure of collision energy. The stress frequency represents the number of all milling bead collisions occurring in 1 s of process time and increases with increasing rotational speed. Summing up the *SF* over a period time results in the overall collision or stress number *SN* (*SN* = *SF* · *t*_process_) respectively that occurred during a certain time of milling. The two parameters $\bar{SE}$ and *SF*, along with the milling time and loaded mass, define the specific energy input into the cathode composite (see Equation 1).

(1) $$E_{m}=\frac{\bar{SE}\cdot SF}{m_{\mathrm{total}}}\cdot t_{\mathrm{process}}=\frac{\bar{SE}\cdot SN}{m_{\mathrm{total}}}$$

### 2.2 Microstructural Analysis of Composites: Visual Observations and Particle Size Measurements

**Figure 2:**
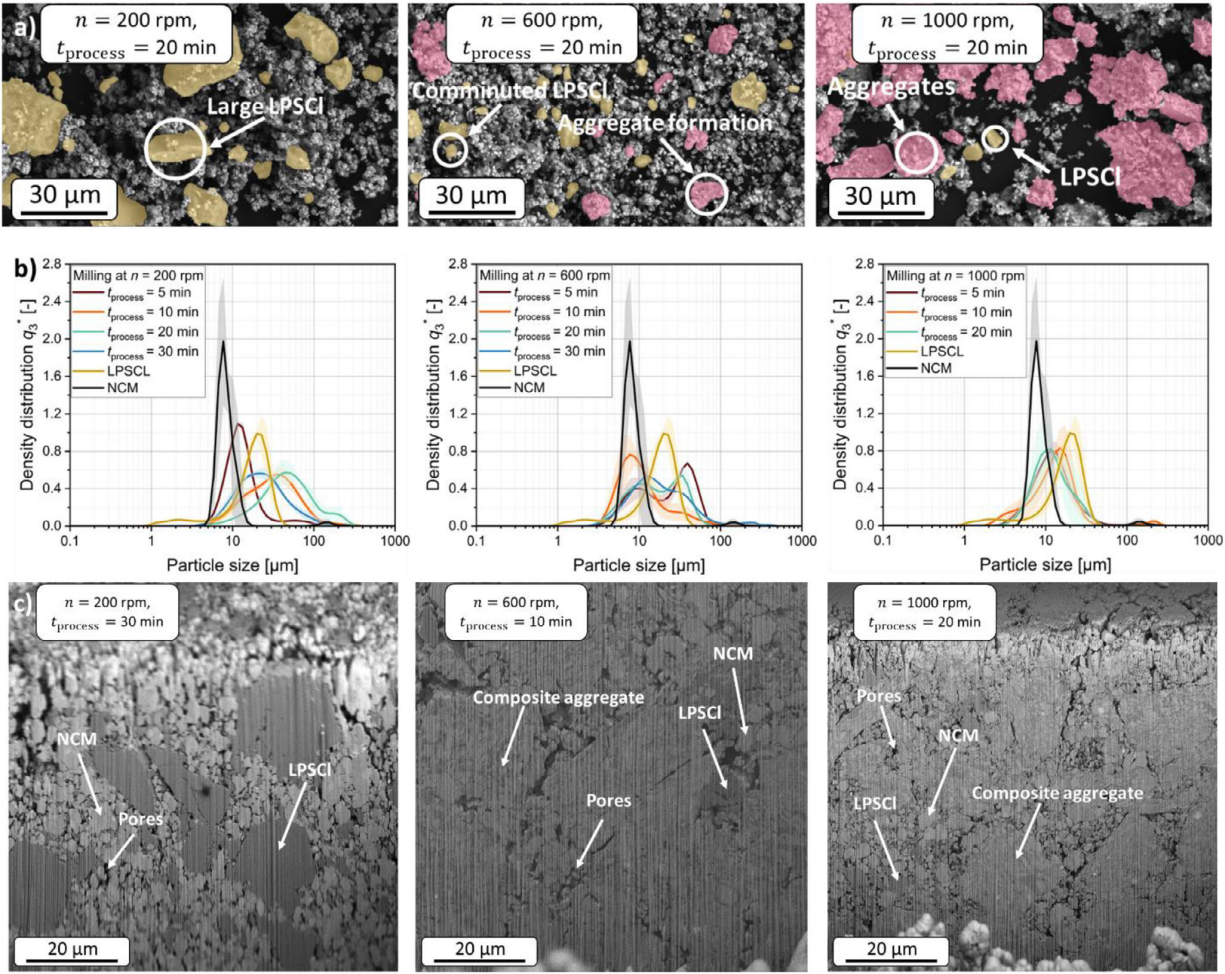
a) SEM images of NCM – LPSCl composite powders processed at different rotational speeds for 20 min. b) Volume‐based particle size distributions of the NCM–LPSCl composites processed at different rotational speeds for 5 to 30 min c) FIB‐SEM images of the pressed cathode composites produced at 200 rpm and 30 min, 600 rpm and 10 min, and 1000 rpm and 20 min of milling.

In **Figure** **2**a,b SEM images and corresponding particle size distributions of the raw materials and cathode composites obtained for the rotational speeds of 200, 600, and 1000 rpm, a milling bead filling ratio of φ = 0.5, and a milling time of 20 min are shown exemplarily. These show that the microstructure of the cathode composite is strongly influenced by the rotational speed, and thus, by the mean stress energy $\bar{SE}$ during the milling bead collisions. At low collision energies (200 rpm), both NCM particles and a large number of big LPSCl particles are evident. This indicates that the stress energy is not sufficient to comminute the LPSCl particles at the given number of collisions. With increasing rotational speed (600 rpm), the number of comminuted LPSCl particles increases, and aggregate particles consisting of NCM and LPSCl become apparent. The comminution of the LPSCl occurs as a result of the increase in collision energy and frequency, which ensures that the LPSCl particles break. In addition, the low hardness of the LPSCl^[^ ^43^, ^44^ ^]^ ensures that it can fuse with the NCM, resulting in aggregated particles from both components. Increasing the rotational speed and thus stress energy and frequency to 1000 rpm results in predominantly aggregate particles of NCM and LPSCl, which is based particularly on the high $\bar{SE}$ during collisions and thus high energy transfer.

Comminution processes are typically characterized effectively by analyzing the particle size of the comminuted material in comparison to the starting material. The median particle sizes (*x*_50_) of the starting materials were measured as 8 ± 2 µm for NCM and 17 ± 2 µm for LPSCl (Figure 2b). For NCM, the measured particle size exceeds the manufacturer‐specified primary particle size of 3–5 µm, indicating that the measured values primarily correspond to agglomerates present in the starting material, which are also visible in Figure 1a.

Laser scattering measurements of particle size in LPSCl‐based cathode composites reveal a complex interplay of comminution and aggregation, complicating interpretation. While comminution reduces the median particle size *x*_50_, aggregation and agglomeration can increase it, leading to a misleading trend. For example, at 200 rpm, *x*_50_ initially rises to ≈44 µm before decreasing again as deagglomeration dominates. At 600 rpm, strong bimodality emerges in the particle size distribution, indicating the coexistence of fine primary particles and larger aggregates, despite *x*_50_ fluctuating moderately over time. This bimodality is also partially evident in the composites processed at 400 rpm (Figure S1a, Supporting Information). With increasing rotational speed, particle size fluctuations decrease, and a stable particle size is observed at 1000 rpm over the entire milling time (see also Figure S1b (Supporting Information) for 800 rpm, Supporting Information).

These overlapping effects make laser scattering unsuitable for accurately tracking LPSCl comminution within composites, as it captures both aggregates and agglomerates alongside primary particles. To overcome this limitation, FIB‐SEM cross‐section imaging was used to directly visualize the composite microstructure and clarify the milling process (Figure 2c). The FIB‐SEM images reveal that at a low rotational speed of 200 rpm, primary particles of NCM and comparatively large LPSCl particles are predominantly present. The latter reach particle sizes of ≈30 µm and are therefore comparable in size to the raw material particles of LPSCl (Figure 1a). This finding is consistent with the observations from the particle size analyses and SEM images, which indicate that no significant comminution or aggregation with NCM occurred at a given rotational speed and milling time. However, when the rotational speed is increased to 600 and 1000 rpm, coarse LPSCl particles are less (600 rpm) or almost no longer present (1000 rpm) in the microstructure. Instead, milled composite aggregates are evident, indicating a fusion of the NCM and LPSCl phases. However, at 600 rpm, some of the pure starting phases are still present, as can be seen from further FIB‐SEM images (Figure S2a,b, Supporting Information). The presence of these many particle species, i.e., pure NCM and LPSCl and a large number of composite aggregates, explains the bimodality of the particle size distribution of the composite produced at 600 rpm (Figure 2b).

Both effects, the comminution of LPSCl and the aggregate formation with NCM, are expected to have a positive effect, especially on the effective ionic conductivity of the composite due to the increase in the number of heterocontacts per volume.^[^ ^8^ ^]^ Since this was achieved in particular at high rotational speeds, it could be concluded that a sufficiently high rotational speed and thus high stress energies are required to optimize the cathode composite. However, it should not be neglected that the bulk conductivity of the electrolyte can decrease due to degradation effects if the stress energies are too high.^[^ ^27^ ^]^ In such a case, it may be necessary to lower the mill's rotational speed. However, this would result in a reduction in the collision number, which could mean that at a given process time, the total energy input is not sufficient to reach the desired cathode composite microstructure.

### 2.3 DEM Simulation of Milling Process: Quantification of Stressing Conditions

To calculate the decisive characteristic parameters stress energy $\bar{SE}$ and stress frequency *SF*, DEM simulations were carried out to optimize the cathode composite formation process and to gain insights into the milling process. Each milling bead experiences a different stress energy *SE* with each collision, and by classifying these different stress energies into discrete intervals, stress frequency‐stress energy (*SF*  −  *SE*) curves were generated that indicate which collision energies occur in 1 s of milling.

**Figure 3:**
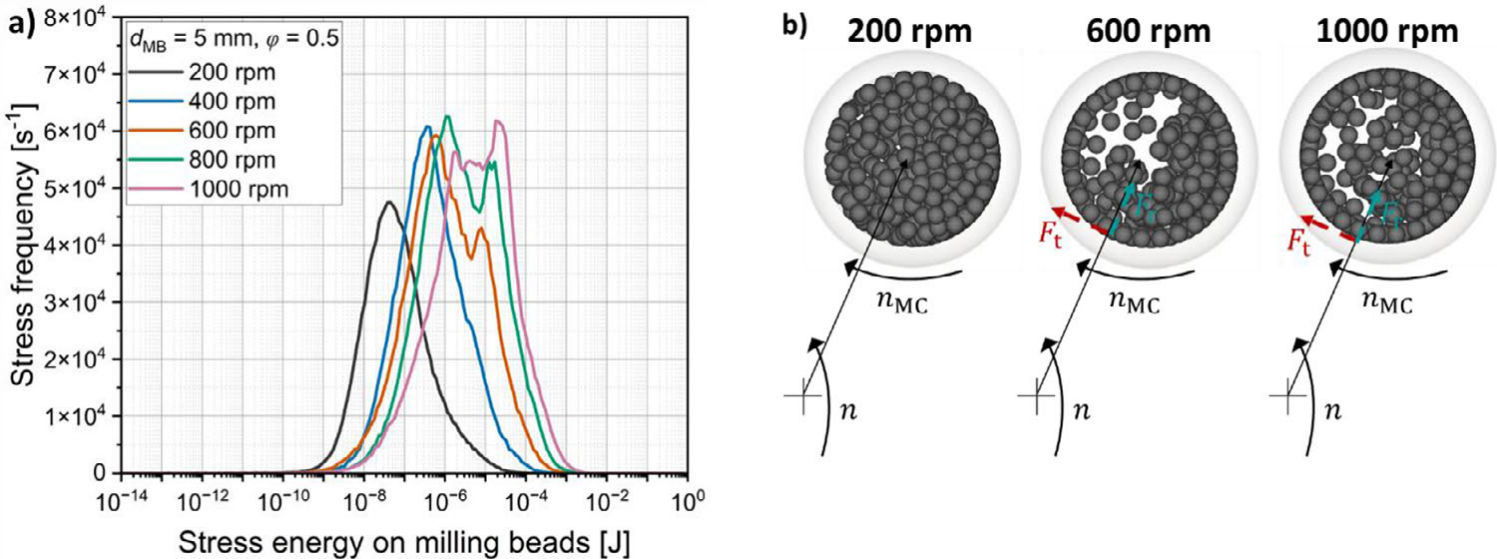
a) Stress frequency–stress energy distribution of milling bead collisions for different rotational speeds. b) Motion pattern of the milling beads in the milling chamber for different rotational speeds.

The *SF*  −  *SE* curves of the different rotational speeds with a milling bead size of 5 mm (**Figure** **3**a) reveal a clear shift toward higher $\bar{SE}$ with increasing rotational speed. This shift was expected as the increased rotational speed leads to a higher relative velocity between the milling beads, and between the milling beads and the milling chamber wall.

The *SF* strongly depends on the motion behavior of the milling beads, which changes significantly with increasing rotational speed due to friction and restitution.^[^ ^45^ ^]^ At lower rotational speeds (200 rpm), the milling beads exhibit predominantly cascading motion (Figure 3b). With an increase in rotational speed to 400–1000 rpm, this motion changes to a combination of rolling at the milling chamber wall and cataracting behavior, in which individual milling beads detach from the wall.^[^ ^46^ ^]^

The motion behavior of individual milling beads in a planetary ball mill is dependent on different forces, namely the centrifugal force due to the discs motion *F*_z_, the centrifugal force of the relative milling chamber motion *F*_z,rel_, the Coriolis force *F*_cor_, and other quantities like the position of the milling beads in the mill and especially friction conditions and filling ratio.^[^ ^45^, ^47^ ^]^ These forces and friction conditions result in a tangential force *F*_t_ that acts in the direction of rotation of the milling chamber and a resulting radial force *F*_r_, which, if it becomes negative, causes the milling beads to detach from the milling chamber wall and thus leads to cataracting behavior. This transition disrupts the initially homogeneous stress energy distribution observed at 200 rpm, resulting in an inhomogeneous distribution with a significantly broader range of collision energies observable in Figure 3a.

The milling result of the NCM – LPSCl composite is largely dependent on the two parameters mean stress energy $\bar{SE}$ and stress frequency *SF*. The stress energy must be sufficient for particle breakage of the LPSCl, and at the same time a sufficiently high stress frequency should be present for further comminution of the fragments or aggregation with each other.^[^ ^40^ ^]^ In laboratories, however, milling beads of different sizes are often present, which can result in a completely different milling result compared to this study (***d***_**MB**_ =  **5 mm,** ***φ*** =  **0.5**). Thus, the ball milling process was also simulated for milling bead sizes of 1.75 and 3 mm and also different filling ratios in order to obtain Equations ((2), (3), (4), (5), (6), (7)) and values for $\bar{SE}$ and *SF* (Table S1–S3, Supporting Information) which should make it possible to determine the energy input required for the cathode composite for a given milling bead size.

(2) $$\bar{SE}\left(\varphi =0.1\right)\propto const\cdot n^{2.31}\cdot d_{\mathrm{MB}}^{3.11}$$

(3) $$SF\left(\varphi =0.1\right)\propto const\cdot n^{0.71}\cdot d_{\mathrm{MB}}^{-3.6}$$

(4) $$\bar{SE}\left(\varphi =0.3\right)\propto const\cdot n^{2.39}\cdot d_{\mathrm{MB}}^{3.86}$$

(5) $$SF\left(\varphi =0.3\right)\propto const\cdot n^{0.62}\cdot d_{\mathrm{MB}}^{-4.21}$$

(6) $$\bar{SE}\left(\varphi =0.5\right)\propto const\cdot n^{2.62}\cdot d_{\mathrm{MB}}^{3.81}$$

(7) $$SF\left(\varphi =0.5\right)\propto const\cdot n^{0.29}\cdot d_{\mathrm{MB}}^{-4.02}$$

**Figure 4:**
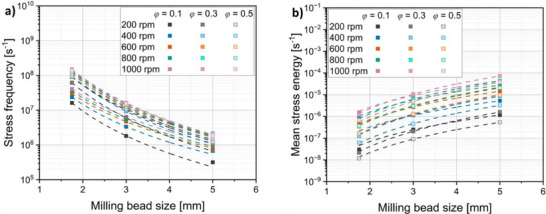
a) Influence of milling bead size on the ***SF*** for different rotational speeds. b) Stress energy dependency on the milling bead size for different rotational speeds and filling ratios.

The simulation results demonstrate that a decrease in the milling bead diameter *d*_MB_ leads to the stress frequency being increased at all rotational speeds and filling ratios by a factor of ≈4 (**Figure** **4**a), which is consistent with the findings of Burmeister et al. for general dry milling in planetary ball mills.^[^ ^48^ ^]^ This means that significantly more collisions occur in the same process time when the same milling time is applied as in this study. On the other hand, the stress energy during the milling bead collisions decreases by a factor of ≈3.8 depending on the rotational speed and filling ratio that is used (Figure 4b).

When a certain milling bead size is available in the laboratory, increasing the rotational speed raises both $\bar{SE}$ and *SF* (Figure S3a,b, Supporting Information). The influence of filling ratio is more complex and less predictable (Supporting Information Figure S4a,b). At low rotational speeds, the *SF* increases with higher filling ratios, but at high rotational speeds (800–1000 rpm), the stress frequency initially rises and then decreases slightly, likely due to milling beads obstructing each other, thus reducing collision frequency. For the mean stress energy, a decrease is observed with increasing filling ratio at low rotational speeds, while at higher speeds, $\bar{SE}$ slightly increases up to a filling ratio of 0.3 before declining again at 0.5.

A parameter that links both variables, the stress energy $\bar{SE}$ and the stress frequency *SF*, is the specific energy input *E*_m_ transferred to the product particles. This denotes how much energy is transferred into the cathode composite by all milling bead collisions during the entire process time and is determined via the mean stress energy $\bar{SE}$, the stress frequency *SF* or stress number *SN*, the process time *t*_process_ and the total mass stressed *m*_total_ (see Equation 1).^[^ ^39^ ^]^

The specific energy input *E*_m_ is a valuable metric for process optimization, as it can help maximize product performance while reducing production time and energy consumption.^[^ ^49^ ^]^ However, it is important to consider that the *E*_m_ is a product of both the mean stress energy and the stress frequency. If the $\bar{SE}$ is too low to achieve effective milling, even a high *SF* and thus a high *E*_m_, may not meaningfully improve the microstructure of the composite.

### 2.4 Influence of Specific Energy Input *E*_m_ on Cathode Composites

**Figure 5:**
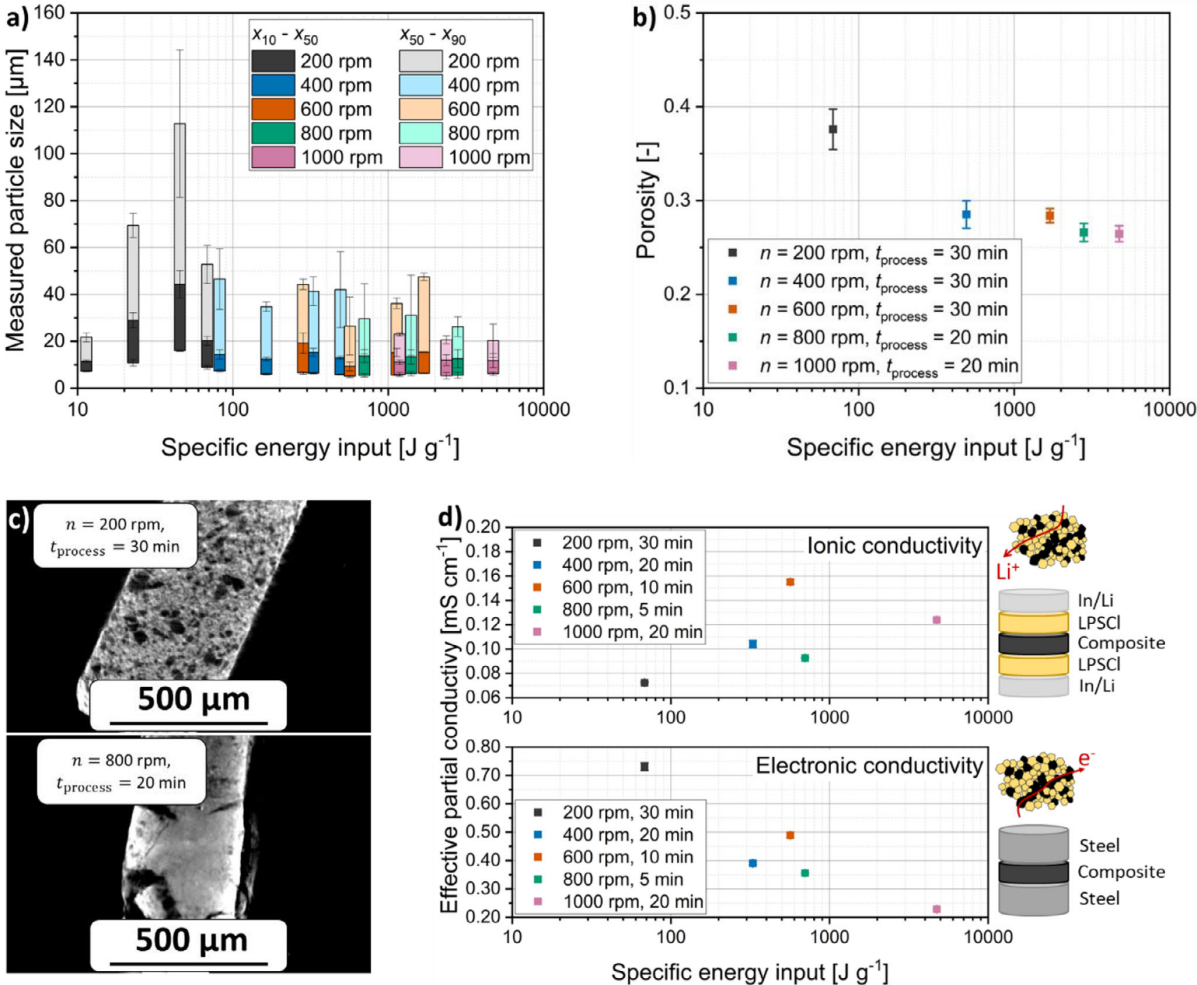
a) Measured particle size as a function of the specific energy input. b) Porosity of compacted cathode composite processed at different rotational speeds. c) µ‐CT scans of the composites processed at 200 rpm for 30 min and 800 rpm for 20 min. d) Effective partial electronic and ionic conductivity of selected composites shown as a function of the specific energy input.

The results presented so far indicate that the planetary ball milling process has a significant impact on the cathode composite of NCM and LPSCl. This is evident from the measured particle size distribution as a function of the specific energy input (**Figure** **5**a). In addition to the median particle size *x*_50_, the interval from *x*_10_ to *x*_50_ and the interval from *x*_50_ to *x*_90_ are also plotted to visualize the influence of the energy input on the width of the particle size distribution.

Initially, at low energy input and thus a low stress energy (200 rpm), the measured particle size *x*_50_ increases due to agglomeration of large LPSCl particles with both LPSCl and NCM. As energy input increases, deagglomeration and comminution of larger LPSCl particles occur, leading to a decrease in particle size. Beyond an energy input of ≈45 J g^−1^ (200 rpm, 20 min), a constant exponential drop in the *x*_50_ is observed with increasing *E*_m_, approaching a limit of ≈10 µm after a specific energy input of 4700 J g^−1^ (1000 rpm and 20 min).

Besides *E*_m_, the stress energy $\bar{SE}$ appears to have a significant impact on the width of the particle size distribution. At low $\bar{SE}$ (200 and 400 rpm), broad particle size distributions are visible. This is attributed to the presence of larger primary LPSCl particles, which are only partially comminuted and tend to agglomerate due to low‐energetic collisions, forming large and uneven agglomerates. In contrast, higher stress energies (600–1000 rpm) lead to the formation of more uniform aggregate particles, resulting in narrower size distributions, especially at rotational speeds of 1000 and 800 rpm. An exception is observed at 200 rpm after 5 min of milling, where the distribution is also narrow, likely because the sample still contains mostly individual raw material particles before agglomeration.

The measured particle size distributions can only be meaningfully interpreted in combination with further analysis. FIB‐SEM images (Figure 2c) confirmed that a comminution of LPSCl and aggregate formation with NCM takes place with increasing stress energy and increasing energy input. Smaller aggregate sizes and the comminution of primary LPSCl particles result in a denser structure of the composite, reducing void content and overall porosity of the composites (Figure 5b) after compaction. Porosity decreases with increasing energy input, dropping from 37.6% to 26.5% after 30 min of milling at 1000 rpm. This improved packing results in better void filling, more uniform microstructures (Figure 5c), and ultimately a higher number of heterocontacts per volume.

The comminution of the solid electrolyte and the resulting reduction in porosity lead to the formation of more continuous ionic conduction pathways within the composite, which is reflected by an increase in effective ionic conductivity with increasing specific energy input (Figure 5d). The maximum ionic conductivity is observed after milling at 600 rpm for 10 min. Beyond this point, i.e., this stress energy, further milling results in a decline in effective ionic conductivity, likely due to degradation of the solid electrolyte and continued reduction in crystallite size.^[^ ^27^ ^]^ At 800 rpm, the drop is more pronounced, probably due to a lower stress number and suboptimal particle arrangement from shorter milling times. In contrast, the effective electronic conductivity of the composite decreases continuously with increasing specific energy input. This trend is attributed to the comminution of the electronically insulating LPSCl, which progressively disrupts the percolation of electronic conduction paths.

**Figure 6:**
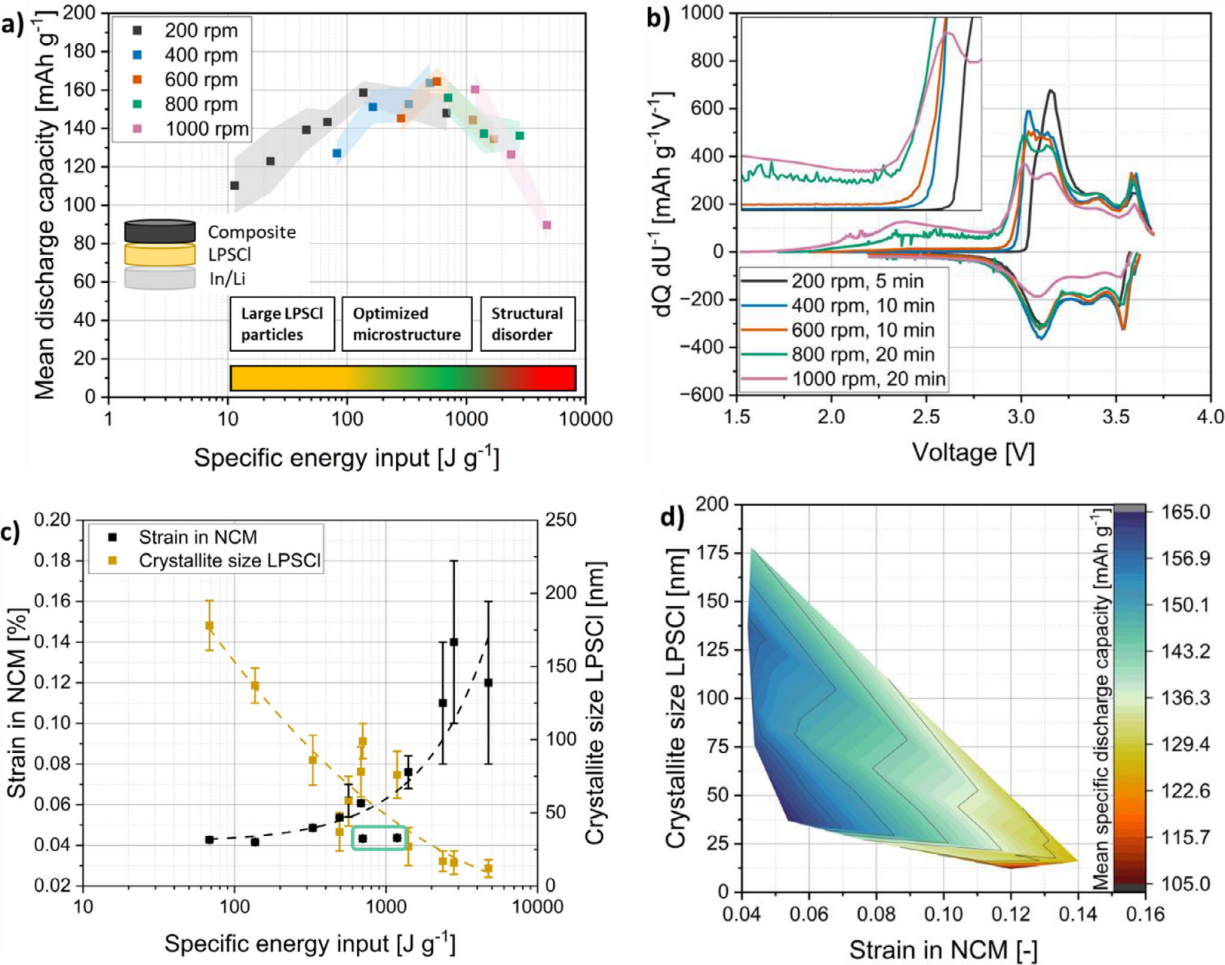
a) Mean discharge capacity of two cells cycled over 20 cycles with the respective cathode composites at 0.1C/0.1C, plotted as a function of specific energy input. b) dQ dU^−1^ diagrams of the first charge and discharge of selected composites. c) NCM strain and LPSCl crystallite size as a function of the specific energy input. d) The mean specific discharge capacity as a function of the LPSCl crystallite size and the NCM strain.

All occurring effects, i.e., the comminution of LPSCl, the deagglomeration of NCM particles from the raw material, the aggregation of NCM and LPSCl, as well as the size reduction of large aggregates, ensure an improvement in the microstructure with increasing energy input *E*_m_ by creating new interfaces and improving ion transport in the composite.^[^ ^21^, ^50^, ^51^ ^]^ This has a positive effect on cell performance (**Figure** **6**a), revealing an increase in the mean discharge capacity of the composites with increasing specific energy input. The mean discharge capacity calculated based on two cells cycled at 0.1C/0.1C for 20 cycles increases from 110 mAh g^−1^ at an energy input of *E*_m_ = 10 J g^−1^ (200 rpm, 5 min) to ≈165 mAh g^−1^ after an energy input of ≈500 ± 100 J g^−1^. This corresponds to milling at 600 rpm for 10 min or 400 rpm for 30 min. In both cases, an optimized microstructure appears to be formed, driven primarily by the specific energy input. To corrobate the electrochemical similarity of these composites, we compared 20 cycles at 0.1C, dQ dU^−1^ curves after 1, 2, and 20 cycles, as well as a C‐rate test, the corresponding dQ dU^−1^ curves at 0.3C and 1C and discharge curves of both composites (Figure S5, Supporting Information). Across all electrochemical measurements, the performance of the 600 and 400 rpm composites closely aligns, indicating that their microstructures are indeed highly comparable.

Despite the significant difference in mean discharge capacity between the composites prepared at 200 rpm for 5 min (110 mAh g^−1^) and 600 rpm for 10 min (165 mAh g^−1^), both show a comparable static cathode active material (CAM) utilization^[^ ^31^ ^]^ of ≈88% (Figure S12b, Supporting Information). This should correspond to a measurable specific capacity of 176 mAh g^−1^, assuming a theoretical specific discharge capacity of 200 mAh g^−1^ for NCM. However, this value is by far not achieved for the 200 rpm composite when cycled at 0.1C/0.1C, indicating a severe kinetic limitation. This limitation is reflected in the differential capacity plots (Figure 6b), which show that the overpotential is significantly higher for the composites prepared at 200 rpm compared to those processed at 600 rpm. Together with the lower effective ionic conductivity, this results in the overall poorer electrochemical performance of the 200 rpm composites.

Composites initially milled at 200 rpm for 30 min did not reach the optimized energy input range of 500 ± 100 J g^−1^. Thus, DEM simulations (Table S1, Supporting Information) were used to estimate the milling times needed to reach this range, which is why additional composites were prepared by milling for 60 and 300 min at 200 rpm. Their cyclization performance confirms that the optimized specific discharge capacity can be achieved, demonstrating that specific energy input is an effective lever to tune the microstructure and enhance cell performance. This also indicates that, with 5 mm milling beads, the stress energy $\bar{SE}$ is not the limiting factor, as long as sufficiently high stress numbers are reached due to long milling times. However, this may not hold for smaller milling beads, where the stress energy might be too low for effective comminution or aggregation. Conversely, larger milling beads can lead to excessively strong collision energies and potentially degrade the composite.

A degrading effect is visible for 5 mm milling beads, when a high specific energy input of > 1000 J g^−1^ is applied (Figure 6a). This is evident from the reduction in the mean discharge capacity of the cycled composites beyond the optimized energy input range. A milling at 1000 rpm and 20 min (4700 J g^−1^) leads to a drop in discharge capacity to ≈90 mAh g^−1^. One reason for this drop in capacity is visible in the differential charging and discharging profiles (dQ dU^−1^ diagram) of the first cycle of the composites (Figure 6b). These show that the composites, which were previously exposed to a higher specific energy input and stress energy, increase the amount of charge induced even at low voltages. This is likely caused by the electrochemical degradation of the LPSCl,^[^ ^52^, ^53^ ^]^ which increases as the composite is stressed during milling. The degradation is most significant in the first cycle (Figure S6, Supporting Information), indicating that a certain amount of LPSCl is oxidized initially and a comparatively stable cyclization is possible afterward. Nevertheless, a remarkable flattening of the charge and discharge curves can be observed in the composites exposed to high energy input. This indicates that a larger proportion of the active material becomes electrochemically inactive.

In contrast, a higher specific energy input and stress energy promote the formation of composite aggregates with improved interfacial contact, resulting in a significantly lower overpotential, especially in the first cycle. This strong difference in overpotential persists throughout subsequent charge and discharge cycles (Figure S6, Supporting Information), particularly for the composite milled at 200 rpm for 5 min, indicating a consistently poorer interfacial contact between the active material and the solid electrolyte.

To quantify the degrading effect of the milling process on the composite, XRD measurements were carried out with subsequent Rietveld analyses. The XRD patterns reveal a broadening of the reflections for both the LPSCl and the NCM with an increase in rotational speed (Figure S7a, Supporting Information), indicating potential structural changes of both, NCM and LPSCl. With the help of Rietveld analyses, a decrease in crystallite size was found for the LPSCl with an increase in specific energy input (Figure 6c). A decreasing crystallite size can indicate a decrease in LPSCl particle size due to comminution.^[^ ^27^ ^]^ This can have a positive effect on the effective ionic conductivity of the composite and thus would result in an enhanced cyclization performance.^[^ ^27^ ^]^ In addition, refining the grain size distribution can lead to a more homogeneous distribution of tension in the LPSCl.^[^ ^24^ ^]^ For the interface between NCM and LPSCl, this would mean an improvement of the physical contact between the active material and the solid electrolyte, which was actually observed in the dQ dU^−1^ diagrams (Figure 6b; Figure S6, Supporting Information). This means that mechanical stresses caused by volume expansion and contraction during cyclization can be compensated for more effectively. However, when the particles are comminuted further and when the crystallite size decreases further, the amount of grain boundaries increases, which can reduce the effective ionic conductivity in the composite,^[^ ^52^ ^]^ as also evidenced for the composites milled at higher rotational speeds (Figure 5d). Especially at high energy inputs, the small crystallite sizes of less than 25 nm could be one reason for the drop in the capacity of the composites that experienced a high energy input due to long milling times and or high rotational speeds.

Additionally, Rietveld analyses revealed that lattice strain in the NCM increases with an increase in specific energy input. High strain in NCM particles is typically detrimental to the composite, as it can lead to fracture, pulverization, and subsequent reactions with the solid electrolyte.^[^ ^54^, ^55^, ^56^ ^]^ A study by Liu et al.^[^ ^57^ ^]^ indicates that crystal defects lower energy barriers for oxygen removal, triggering Li^+^ and transition metal ion mixing in NCM, ultimately causing structural degradation. We hypothesize that the increased strain arises from lattice defects in the NCM crystal structure, induced by high‐energy milling bead collisions, similar to findings with tin oxide particles.^[^ ^58^ ^]^ This strain increases with increasing specific energy input, ultimately leading to degradation of NCM particles and reduced electrochemical performance. However, two cathode composites produced at 800 rpm for 5 min and 1000 rpm for 5 min do not follow this trend (marked in light green), as despite the higher specific energy input, the strain in the NCM is not increased. This can be attributed to the low stress number, which, after 5 min is still significantly lower than that of the other composites (Figure S7b, Supporting Information). In contrast, the sample milled at 200 rpm for 300 min exhibits the highest stress number, but shows a significantly lower strain compared to samples milled for 20 min at 800 or 1000 rpm. This suggests that the mean stress energy at 200 rpm is not sufficient to strain the NCM as strongly as the higher mean stress energies at 800 and 1000 rpm. Therefore, not only the total energy input, but also its individual components, stress number, and stress energy, must be considered separately to fully understand their impact on the cathode composite microstructure.

The dQ dU^−1^ plots alongside the Rietveld analyses indicate that significant degradation occurs during the first cycle, particularly in the highly stressed composites. To confirm this, a postmortem analysis was conducted for a highly stressed (1000 rpm, 20 min) composite. As already observed in (Figure 6b), the differential capacity plots highlight the pronounced impact of milling, with the highly stressed composite exhibiting clear signs of severe degradation during the first cycle and even stronger degradation after 20 cycles (Figure S8a, Supporting Information). Additionally, Raman spectroscopy reveals notable peak broadening compared to pure LPSCl, both after composite formation and, more prominently, after one cycle at 0.1C (Figure S8b,c, Supporting Information). This broadening suggests structural changes in the LPSCl, likely related to degradation occurring during the initial cycling.

Finally, volume changes during lithiation and delithiation can result in performance losses if they are not effectively accommodated. The postmortem analysis of the composite milled at 1000 rpm for 20 min clearly reveals the formation of cracks within the aggregate particles after just the first cycle (Figure S8d, Supporting Information). After 20 cycles at 0.1C, extensive cracking is observed throughout the composite structure. These cracks arise from insufficient mechanical accommodation to buffer the repeated volume expansion during cycling, leading to progressive contact loss and, consequently, a further decline in electrochemical performance.

To bridge the gap between process influence on the microstructure (crystallite size and strain) and thus discharge capacity (property), the mean discharge capacity is displayed over crystallite size and NCM strain in Figure 6d. This clearly shows that an increasing NCM strain has a negative effect on the discharge capacity and should therefore be minimized. For the crystallite size, a decrease up to a size of 35 to 40 nm should be aimed for, as here the highest mean discharge capacities could be achieved.

### 2.5 Using Cathode Composites for Upscaled Process Routes: Dry Film Production

**Figure 7:**
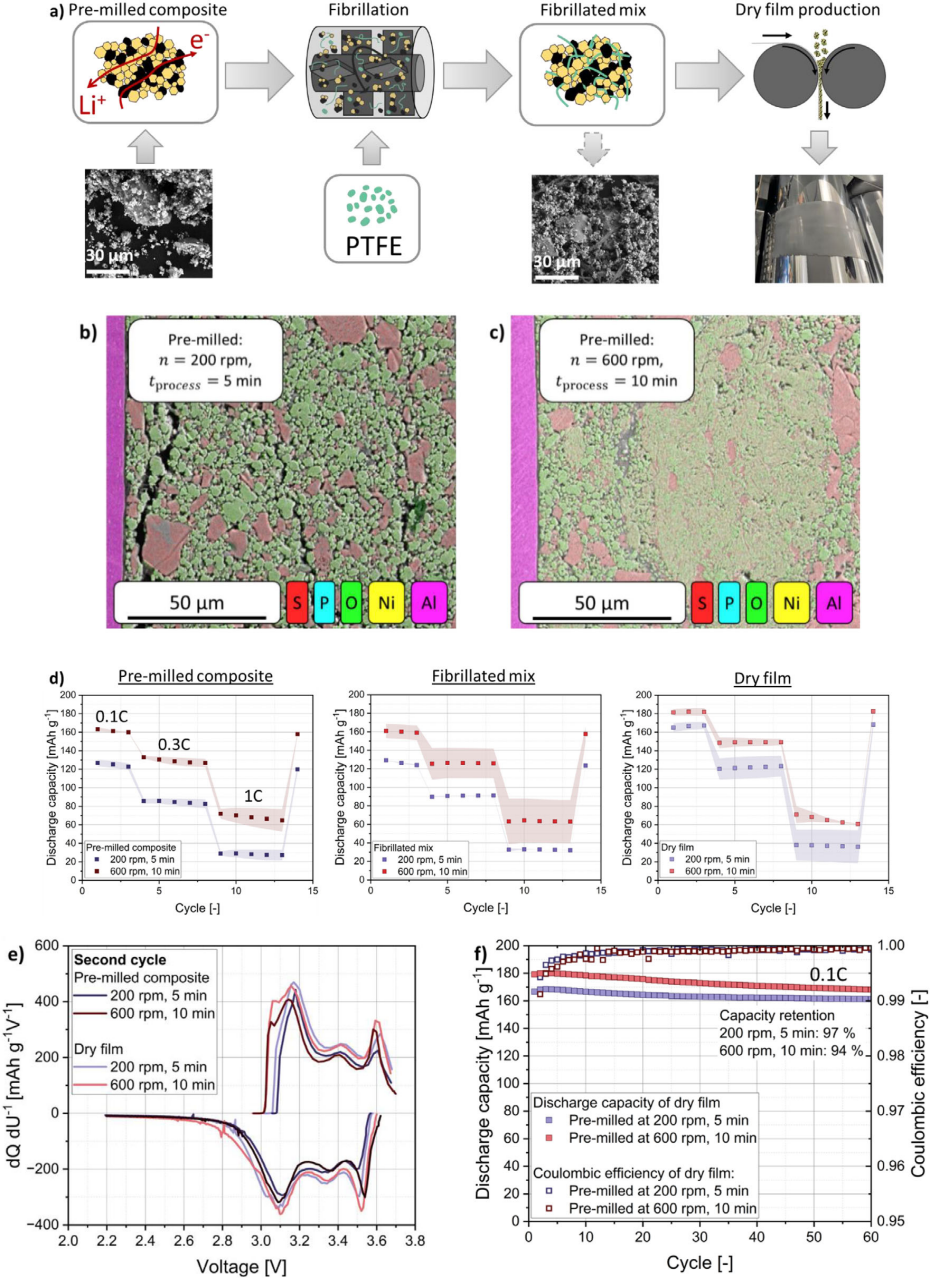
a) Schematic representation of the fibrillation and dry film production step. b) SEM cross‐section image with EDS mapping of the dry produced film for which the composite was pre‐milled at 200 rpm for 5 min. c) SEM cross‐section image with EDS mapping of the dry produced film for which the composite was pre‐milled at 600 rpm for 10 min. d) C‐rate tests for two pre‐milled composites: The cathode composite prepared at 600 rpm and 10 min, and the cathode composite prepared at 200 rpm and 5 min, as well as their further mixture with PTFE and the dry produced electrode from these mixtures. e) dQ dU^−1^ diagrams of the second charge and discharge of pre‐milled composite and dry films. f) 60 cycles at 0.1C of the dry produced electrodes with dry films previously milled at 200 rpm and 5 min, as well as 600 rpm and 10 min.

The optimized cathode composites from milling at 600 rpm for 10 min (best cell performance at 0.1C) and the one milled for 5 min at 200 rpm (poor cell performance at 0.1C) were further processed to show the influence of pre‐milling on the large‐scale processing of SSBs. In the first step, 3 wt.% PTFE binder was added to the cathode composite, and the mixture was stressed in a high‐intensity ring‐shear mixer, resulting in a successful fibrillation of the binder (**Figure** **7**a). Subsequent calendering of the PTFE–LPSCl–NCM mixture led to a homogeneous film formation on the aluminum current collector with a mass loading 30 mg cm^−2^ and a porosity of ɛ  = 0.3 for both films.

SEM images and EDS mapping of ion‐polished cross sections of the dry produced films (Figure 7b,c) show that, as with the pure composites, primary particles of LPSCl and NCM are present at low energetic pre‐milling of 200 rpm and 5 min. On the other hand, the dry produced film for which the composite was pre‐milled at 600 rpm and 10 min contains composite aggregates in which the two phases appear fused. This fusion process can also result in the NCM particles being coated with a thick layer of the solid electrolyte. Although this is not observed in the present case, as confirmed by the EDX images, such a coating could potentially enhance electrochemical performance by improving interfacial contact between active material and electrolyte.^[^ ^59^, ^60^ ^]^ High‐intensity mixing techniques, which induce strong shear forces, are suitable for generating such coatings in a controlled manner.^[^ ^61^, ^62^, ^63^, ^64^ ^]^ However, in this case, the addition of a conductive additive is necessary, in order not to shield the NCM particles from the electronic network.

C‐rate tests of the pure NCM – LPSCl composites milled at 600 rpm and 10 min, as well as 200 rpm and 5 min confirm the trend that, also at higher C‐rates of 0.3C and 1C a better performance is recorded for the composite prepared at 600 rpm (Figure 7d). In both cases, the addition of 3 wt.% PTFE to the composite does not have a negative effect on the C‐rate performance, as can be seen from the fact that the specific discharge capacity of the NCM‐LPSCl‐PTFE powder mixtures does not decrease compared to the powder cells prepared with the pre‐milled composites. In fact, a slight stabilization of the discharge capacity can be observed due to the addition of the binder. This is presumably due to the fibril‐like structure of the PTFE, which improves the mechanical integrity without blocking ionic or electronic transport paths.^[^ ^35^ ^]^ FIB‐SEM cross‐sections of dry films containing PTFE revealed particle species and morphologies similar to those observed in the sole cathode composites without PTFE (Figure 7b,c). Together with the minor differences observed in electrochemical performance, this indicates that the pre‐milled composite undergoes minimal structural alteration during the fibrillation step. Therefore, the influence of this step on the pre‐milled cathode composite can be considered negligible in this case. Nevertheless, the distribution and architecture of the PTFE fibrils can significantly impact the final electrode structure and warrant thorough investigation in future studies.

Finally, examining the C‐rate performance of the dry‐produced films shows improved performance at 0.1C, notably with 1.5‐times increase mass loading (30 mg cm^−2^), compared to the pre‐milled composite and the fibrillated mix. The improvement is attributed to the shear stress effect, which acts in the narrow gap during calendering.^[^ ^35^ ^]^ The high shear stresses during calendering compress the particles and promote the formation of new heterocontacts between NCM and LPSCl. This incorporates more NCM particles into the ion‐conducting network, thereby increasing the specific discharge capacity of the calendered films. This improvement is also reflected in the dQ dU^−1^ diagrams, which show a reduced overpotential, particularly for the dry film produced from the 200 rpm composite (Figure 7e). The lower overpotential indicates enhanced interfacial contact between NCM and LPSCl, explaining the performance boost at 0.1C of the dry film compared to the composite.

Enhanced interfacial contact, along with a greater number of particles incorporated into the conducting network, leads to increased CAM utilization. In our study, CAM utilization rose from 88% for both pre‐milled composites to ≈99% after film formation from the 200 rpm, 5 min composite, and to 96% for the dry film based on the 600 rpm pre‐milled composite (Figure S12b, Supporting Information). While the increase in CAM utilization enhances the electrochemical performance at low C‐rates, reaching capacities of 180 mAh g^−1^ (pre‐milled at 600 rpm) and 165 mAh g^−1^ (pre‐milled at 200 rpm), the improvement is hardly or no longer visible when going to higher C‐rates of 0.3C and 1C (Figure 7d). This is due to the fact that the CAM utilization is only a static property, which does not reflect the kinetics of the active particles.^[^ ^31^ ^]^ In the investigated electrodes, large LPSCl particles remain present (Figure S9a,b, Supporting Information) and despite the shear stress effect, the structure obtained from pre‐milling is not significantly changed during subsequent processing. As a result, a comparable tortuosity is still present in the film and powder cells, i.e., a similar secondary degree of mixing,^[^ ^31^ ^]^ which also leads to a similar capacity retention at high charging and discharging currents.

The dry produced film shows that both electrodes perform stably over 60 cycles at 0.1C/0.1C (charging/discharging) (Figure 7f). The film in which the cathode composite was previously stressed at 600 rpm shows the slightly higher specific discharge capacity of 180 mAh g^−1^, as before. However, this electrode exhibits slightly stronger capacity fading, with a retention of 94%, compared to 97% for the electrode for which the cathode composite was previously processed at 200 rpm. This might be due to the fact that at 600 rpm, there are more heterocontacts between the NCM and LPSCl, which amplifies interfacial degradation effects.^[^ ^65^, ^66^, ^67^, ^68^, ^69^ ^]^ In addition, the smaller crystallite size in LPSCl, and thus the higher number of grain boundaries, could enhance this degradation effect. Lastly, the influence of the lithium‐indium anode during long‐term cycling should not be neglected, which can also affect the discharge capacity.^[^ ^70^ ^]^

**Figure 8:**
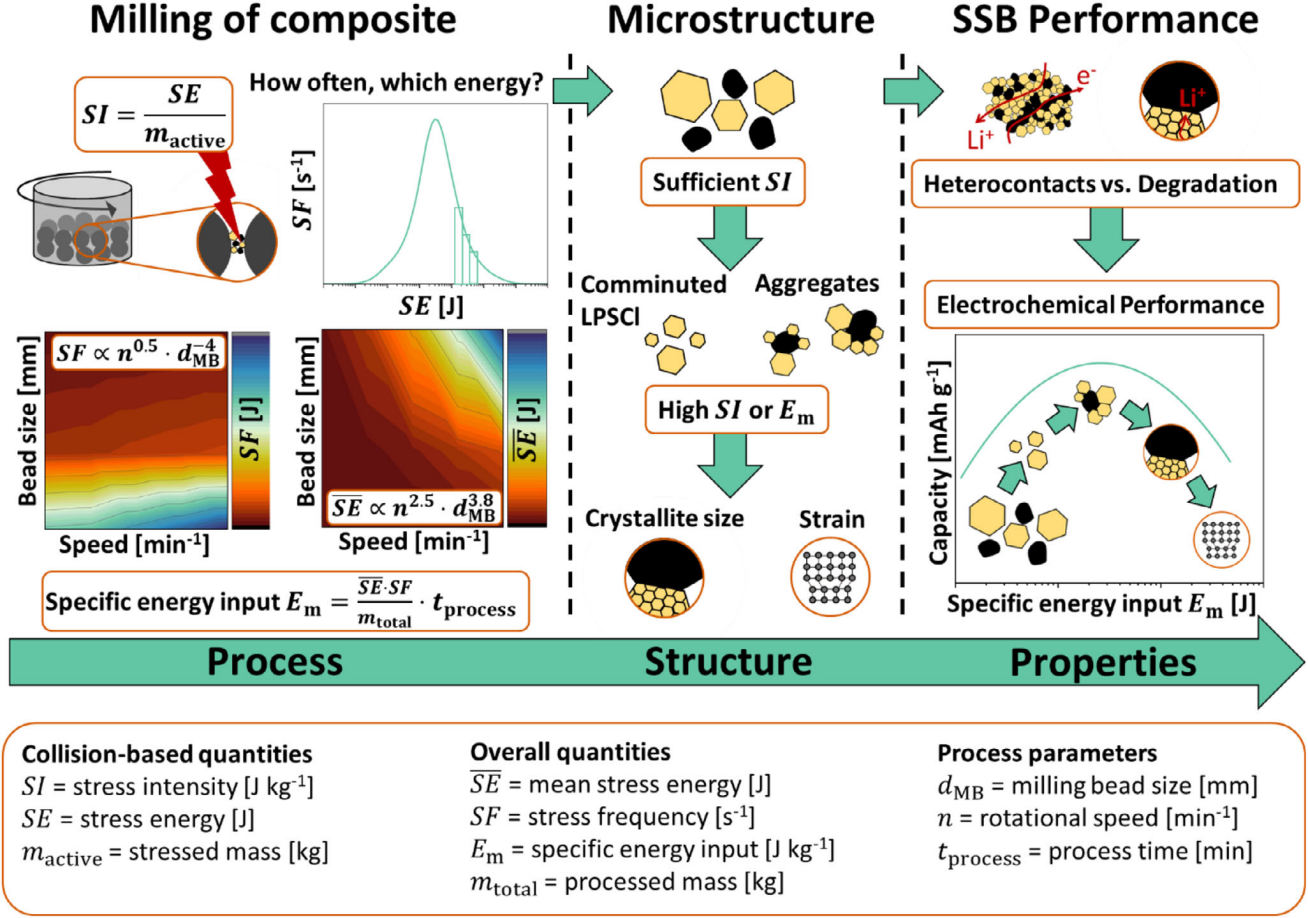
Process–Structure–Property relationships in cathode composite formation: Planetary ball milling strongly influences the microstructure of the cathode composite through variations in rotational speed, milling time, and thus stress energy, frequency, and specific energy input. Key microstructural changes include the comminution of LPSCl, aggregation with NCM, reduced LPSCl crystallite size, and increased lattice strain in NCM. These structural modifications directly affect electrochemical performance. Enhancing effective conductivity and heterocontacts can lead to higher specific discharge capacities, while degradation mechanisms from excessive stress may reduce capacity.

Overall, the electrochemical performance of the dry‐produced cathode films highlights the critical role of an optimized pre‐milling process in enhancing SSB performance. During this process, an improved cathode microstructure needs to be created, leading to enhanced SSB performance. Our findings demonstrate a strong correlation between processing parameters, microstructural changes, and resulting properties in the milling of NCM–LPSCl composites (**Figure** **8**). In particular, stressing conditions such as mean stress energy $\bar{SE}$, stress frequency *SF*, and specific energy input *E*_m_ play a decisive role in this process – structure–property relationship. Still, there remains potential for further optimization, especially in the dry film production of electrodes from the pre‐milled composite. As shown in previous studies, conductive additives can markedly improve electrochemical performance, especially at higher C‐rates.^[^ ^71^, ^72^ ^]^ However, the type of conductive additive used can also increase electrolyte degradation, making careful compositional balancing necessary.^[^ ^72^, ^73^ ^]^ Besides, the dry produced films still possess a comparatively high porosity of ≈30%. This results in higher tortuosity for lithium ion and electron transport paths, thus resulting in a decrease in discharge capacity at high C‐rates. Future work should therefore focus on further densification of the dry produced films by higher roller forces or additional densification steps to further increase the amount of heterocontacts per volume. Lastly, for the cathode films on an industrial scale, large quantities of material are required, which cannot be produced with planetary ball mills. Therefore, this milling process should be transferred to scalable mills in the future. This should be possible by knowing the specific energy input *E*_m_, the mean stress energy $\bar{SE}$ and the stress frequency *SF*. For the transfer, in particular, the $\bar{SE}$ should be the same as in this investigation as this is the crucial parameter influencing the LPSCls crystallinity and the NCM strain whereby the *SN* can be increased by just increasing the milling time to reach the desired specific energy input.

## 3 Conclusion

This study demonstrates that planetary ball milling significantly influences the microstructure of NCM‐LPSCl cathode composites, ultimately affecting the subsequent processing step of dry electrode formation, in SSB production. We show that the formation of different particle species, primary particles of NCM and LPSCl, as well as aggregates of both, depends on stress energy $\bar{SE}$, number *SN* and specific energy input *E*_m_. Higher energy inputs lead to an increased formation of composite aggregate particles and LPSCl comminution, resulting in reduced porosity and a narrower particle size distribution.

A reduction in LPSCl particle size, lower porosity, and the formation of composite aggregates enhance heterocontact formation between NCM and LPSCl, improving electrochemical performance up to a milling specific energy input of ≈500 ± 100 J g^−1^. However, excessive stress energies and a high energy input lead to a reduction in crystallite size within the LPSCl particles and an increase in strain in the NCM, both of which result in a decrease in electrochemical performance at higher energy inputs.

Further processing of pre‐milled composites reveals that planetary ball milling also influences dry‐produced electrode films fabricated with PTFE. Optimized pre‐milling enhances electrochemical performance, particularly at higher C‐rates, while poor pre‐milling can only be partially compensated by high shear stresses during calendering.

Overall, this study underscores the critical role of processing in shaping the microstructure and electrochemical performance of SSB cathode composites. Beyond material innovation, future research should emphasize process optimization and parameter control to advance scalable SSB fabrication. A more interdisciplinary approach, combining material science and chemical process engineering, is essential for accelerating SSB development.

## 4 Experimental Section

### Milling of Composite Cathodes

Cathode composites of NCM (MSE Supplies LLC) and fine LPSCl (NEI Corporation) were produced in a planetary ball mill (Fritsch GmbH, Pulverisette 7). A milling bead filling degree φ of 50% was selected, whereby yttrium‐reinforced milling beads with a diameter of 5 mm were used. Both milling chambers were loaded with 12.25 g NCM and 5.25 g LPSCl, resulting in a mass ratio of 70:30 (NCM:LPSCl) and a volume ratio of 50:50 (NCM:LPSCl). Given the true densities of LPSCl (1.87 g cm^−3^) and NCM (4.75 g cm^−3^), this corresponds to a filling degree of 33.7% between the milling beads. Due to the higher tap densities of both materials, a higher filling level of ≈100% was present in the voids before milling.

For the milling process, the rotational speed of the sun wheel *n* was varied between 200, 400, 600, 800, and 1000 rpm, and a speed ratio *k* between the rotational speed of the sun wheel *n* and the rotational speed of the milling chambers *n*_MC_ was $k=\frac{-n_{\mathrm{MC}}}{n}=-2$. Samples of the cathode composite were, in most cases, taken after 5, 10, 20, and 30 min of milling, whereby sampling and filling of the milling chambers took place in gloveboxes under an argon atmosphere (H_2_O < 1 ppm, O_2_ < 1 ppm). For 200 rpm, also milling times of 60 and 300 min were applied, and for 800 and 1000 rpm, only 20 min of milling were used as further milling resulted in strong heat development and degradation of the LPSCl.

### DEM Simulation of Milling Process

To simulate the planetary ball mill process using DEM, the milling bead properties under the effect of the cathode composite were calibrated as already described in previous studies^[^ ^45^, ^74^, ^75^, ^76^ ^]^ For this, the milling beads covered with material were taken from the composite formation process (600 rpm, 5 min) and then examined by means of a drop‐down test, rolling friction test and static angle of repose test.

For the drop‐down test, individual milling beads were dropped onto a layer of the cathode composite from a defined height, and the velocity before and after the impact was determined via a high‐speed camera in order to determine the restitution coefficient of the material‐covered milling beads (Figure S10a, Supporting Information).

(8) $$e_{\mathrm{MB}}=\frac{v_{2}}{v_{1}}$$

As the coefficient of restitution is strongly dependent on the powder bed height that the milling bead hits, different heights of cathode composite powders were investigated. In total, 20 tests with an average powder bed height of 0.94 mm were performed, from which a mean restitution coefficient of *e*_MB_ = 0.15 was determined.

For determining the coefficients of rolling friction µ_r_ and dynamic friction µ_d_, a milling chamber containing the cathode composite sample from the experiments was used. A milling bead was released from a specified height in the milling chamber, initiating a rolling motion (Figure S10b, Supporting Information). The coefficient of rolling friction was iteratively adjusted to 0.045 and µ_d_ was calibrated to 0.5 by measuring the height of the pendulum‐like motion of the milling bead and comparing these measurements with results from a DEM simulation of the same setup.

Last, the static friction of the milling beads was determined by measuring the static angle of repose of the milling beads (Figure S10c, Supporting Information). For this purpose, a cylinder was used, which was pulled up, and the angle of repose of the milling beads was determined. By comparing the experimentally and simulatively determined slope angle, the static friction of the milling beads was calibrated to µ_s_ = 0.55. The milling beads specific density of ρ  =  6.1 g cm^−3^ and young's modulus of *E*  = 210 GPa were taken from the manufacturer's specification.

The DEM simulation of the milling process was carried out using Rocky 2024. The calibrated milling beads were used, and the process was simulated at rotational speeds of 200, 400, 600, 800, and 1000 rpm for 1 s of milling time. The mean collision power data per milling bead $P_{i}^{\alpha }$ per output interval Δ*t* = 0.5 ms occurring in the simulated time of 0.5–1 s, as well as the collision frequency of the respective milling bead *c*_i_ were exported and converted into collision‐based mean stress energies per milling bead ${\bar{SE}}_{\mathrm{MB}}^{\alpha }$.^[^ ^8^, ^27^ ^]^

(9) $${\bar{SE}}_{\mathrm{MB}}^{\alpha }=\frac{P_{i}^{\alpha }}{c_{i}}=\frac{\sum_{c=1}^{N_{c,i}}W_{c}^{\alpha }}{\Delta t_{\mathrm{out}}}\cdot \frac{\Delta t_{\mathrm{out}}}{N_{c,i}}$$

Here, $W_{c}^{\alpha }$ denotes the collision energy, which is calculated during each collision via contact forces and *N*_c,i_ is the number of collisions for milling bead i during the output time of 0.5 ms. The exponent α stands for the different parts of the collision energy, namely normal, shear, and dissipation. To take into account the collisions occurring in the time interval Δ*t*, the number of collisions occurring per milling bead per time interval was used to determine the stress frequency–stress energy curves (*SF*  −  *SE*),^[^ ^8^ ^]^ which were further used for calculating the specific energy input *E*_m_ that is brought into the material.^[^ ^39^, ^40^, ^41^ ^]^

### SEM, FIB‐SEM, µ‐CT Analysis

A SEM Phenom XL (Thermo Fisher Scientific Inc.) was used for SEM analysis of the stressed composites and raw materials. Imaging was performed at a beam voltage of 15 kV and a pressure of 0.1 Pa in the sample chamber. The SEM device was placed in a dry room with a dew point of the supply air of *T_D_* = − 27 °C. The SEM images were further analyzed with our in‐house *particleOS.ai* tool for particle detection and analysis.

The microstructure of the composite powders without PTFE was investigated by means of focused ion beam (FIB) and SEM imaging using the Xe‐plasma FIB XEIA3 (TESCAN, Czech Republic). For this purpose, ≈100 mg of the powders were uniaxially pressed into pellets with a normal stress of 380 MPa. Large‐area cross sections for EDS mapping of the dry produced electrode films were prepared by cutting them by hand, followed by ion‐polishing at − 100 °C with a triple ion beam cutter (EM TIC 3X, Leica Microsystems, Germany) equipped with three Ar^+^‐ion guns. The operating conditions were 6 kV and a current of ≈2 mA. The polished cross‐sections were imaged using a field‐emission SEM GeminiSEM 560 system (Carl Zeiss Microscopy GmbH, Germany). EDS mapping was performed with the AzTec EDS system (Oxford Instruments, United Kingdom) using the Ultim Max detector. All sample transfers were conducted under vacuum or inert Ar‐atmosphere using the transfer system Leica EM VCT500 (Leica Microsystems GmbH, Germany).

X‐ray measurements were carried out to obtain 3D information on the microstructure of the cathode composites. For this purpose, 100 mg of the composite was pressed by a two‐column lab press (PW 10, Paul‐Otto Weber GmbH) at 380 MPa for 1 min. From the resulting pellets, a fragment with a diameter of 1 mm was broken out and placed in a polyimide tube (inner diameter = 1 mm, length = 10 mm) and sealed at both ends with epoxy resin.

Micro‐computed tomography (µ‐CT) measurements were performed using a MicroXCT‐400 (Xradia). Imaging was performed with a 20× objective providing a pixel size of 0.5992 µm. The X‐ray source was operated with an accelerating voltage of 80 kV and a power of 10 W. Each projection was acquired with an exposure time of 23 s, and a total of 3000 projections were collected.

### Porosity Calculation

The porosity of the composites was determined by pressing 100 mg pellets at 380 MPa for 1 min by a two‐column lab press (PW 10, Paul‐Otto Weber GmbH). From these, the pellet density ρ_pellet_ was determined from the height and weight, and the porosity was determined with the pellet density, as well as the pure mixing density ρ_0_ = 3.25 g cm^−3^ according to Equation (10).

(10) $$\varepsilon =1-\frac{\rho_{\mathrm{pellet}}}{\rho_{0}}$$

For the determination of the porosity of the dry produced films, samples with a diameter of 9 mm were punched and weighted by a fine balance (BCE224i‐1S Entris II, Satorius Lab Instruments GmbH & Co. KG), and the thickness was measured by a tactile gauge (Mitutoyo). The porosity was calculated using Equation (10) and a mixing density of ρ_0_ = 3.20 g cm^−3^ under consideration of PTFE binder.

### Particle Size Analysis

The particle sizes of the cathode composite powder were determined using laser diffraction (Horiba LA‐960). For this, the cathode composite sample was dispersed in p‐xylene (Merck KgaA) in a tube and then measured in a Horiba cuvette device. The refractive index for the LPSCl was used for all measurements. Three volume‐based particle size distributions were determined for each composite sample, and the mean was calculated. In this study, the logarithmic volume‐based density distribution $q_{3}^{*}\left(x\right)$ is shown. In Equation (11), the relation to the volume‐based distribution *q*_3_(*x*) is presented with *x*_up,i_ as upper inerval boundary and *x*_low,i_ as lower interval boundary.^[^ ^77^ ^]^

(11) $$q_{3}\left(ln\;x\right)=q_{3}^{*}\left(x\right)={\left(x_{\mathrm{up},i}\cdot \;x_{\mathrm{low},i}\right)}^{0.5}\cdot \;q_{3}\left(x\right)\cdot \;\frac{\ln10}{1000}$$

### X‐Ray Powder Diffraction (XRD) and Rietveld analyses

X‐ray diffraction measurements were carried out on a Stoe StadiP powder diffractometer in Debye Scherrer geometry with Mo Kα1 radiation (λ = 70.930 pm). The 2θ collection ranged from 4° to 70°, with a step size of 0.015° and a counting time of 90 s per step. For statistics optimization, 5 so obtained patterns were summed up. To avoid material degradation in air, samples were prepared and sealed in borosilicate glass capillaries in an inert (Ar) atmosphere (O_2_ < 0.1 ppm and H_2_O < 1 ppm).

Rietveld refinements were performed using the TOPAS‐Academic V7.25 software package.^[^ ^78^ ^]^ The structural information of LPSCl, taken from Gautam et al. was used as a starting model for this study.^[^ ^79^ ^]^ Structural information of NCM was taken from Lüther et al. to define the starting model.^[^ ^80^ ^]^ The peak shape was described by a pseudo‐Voigt function using the modified Thomson–Cox–Hasting modification.^[^ ^81^ ^]^ Fit indicators *R*_wp_, *R*_exp_, and goodness‐of‐fit were used to assess the quality of the refined structural model. Due to the high collection time and great statistics of the diffraction data, obtained goodness‐of‐fit values obtained range between 2 and 3. First, a refinement of a LaB_6_ standard was carried out to determine the instrumental broadening and to separate processing influences better. The following parameters were refined in the given order: 1) scale factor, 2) background fit by a Chebychev polynomial with 12 parameters, 3) zero error, 4) lattice parameter, and 5) the peak shape. Once a suitable profile fit was achieved, the 5) isotropic thermal displacement parameters, 6) fractional atomic coordinates, and 7) occupancies were refined for all relevant sites. Finally, boron positions were investigated. The so obtained peak shape parameters were applied to and kept constant in all following refinements. Subsequent fitting of powder samples was carried out in an analogous manner, except that only pkw was refined for the peak shape. Additionally, in a last step, the generalized strain and crystallite size were refined according to Balzar et al.^[^ ^82^ ^]^ The fits and the resulting structural parameters from Rietveld refinements against X‐ray powder diffraction are given in Figure S11 and Tables S4–S6 (Supporting Information).

### Cell Assembly and Electrochemical Characterization

Cells consisting of cathode composite, LPSCl separator, and a lithium–indium anode were built for electrochemical characterization. First, 80 mg of the LPSCl was pressed at 380 MPa for 1 min by a two‐column lab press (PW 10, Paul‐Otto Weber GmbH). Cathode composite (15 mg) was spread on the pressed LPSCl separator and pressed at 380 MPa for 1 min. An indium punch (9 mm, Thermo Fisher Scientific Inc.) and a lithium punch (9 mm, Toyota Tsusho Europe) were placed on the other side of the separator. A defined stress of ≈80 MPa was applied in a cell press apparatus (Figure S12a, Supporting Information) to compensate for volume expansion in the composite. In the case of the calendered film, a 9 mm punch was used. The composites and calendered films were tested using two different electrochemical tests, a C‐rate test at 0.1C/0.1C, 0.3C/0.3C, and 1C/1C, as well as a cyclization test for 20 cycles at 0.1C/0.1C (charging/discharging, all CC). In each case, a minimum of two cells was built. All cyclization tests were performed using a BaSyTec XCTS system within a potential range of 2.2 and 3.7 V versus In/InLi at 25 °C.

The active mass determination was carried out in congruently to Kissel et al.^[^ ^31^ ^]^ Reference electrodes of polyvinylidene fluoride (PVDF), CB, and NCM (PVDF:CB:NCM | 3:2:95) were prepared for this purpose. For electrode production, the powders were initially gently dispersed in N‐methyl‐2‐pyrrolidone (NMP) at low circumferential speed, followed by intensive dispersion at 9 m s^−1^ for 1 h with a dissolver (CA40 VMA‐Getzmann GmbH) using a 30 mm dissolver disc. The slurry with a solid content of 70 wt.% was then degassed for 15 min to ensure homogeneity. Coating was performed on 20 µm thick aluminum foil using a batch lab coater (ZAA 2300, Zehntner) with a coating speed of 15 mm s^−1^, a temperature of 80 °C, and a 150 µm doctor blade, resulting in an electrode layer of 57 µm and a density of 1.98 g cm^−3^ (mass loading ≈ 11 mg cm^−2^). To enhance electronic conductivity, the electrode was calendered using a two‐roll calender (Saueressig GK300L, Matthews Europe GmbH), reaching a final density of 2.7 g cm^−3^ and a coating thickness of ≈40 µm. The processes were carried out in a dry room with a supply air dew point of *T*_D_ = − 60 °C. Stamps with a diameter of 14 mm were punched from the reference electrodes to assemble CR2032‐type coin cells against lithium metal (500 µm thick, 15 mm punch, Rockwood Lithium) in a glovebox. As a separator, a porous glass fiber punch (16 mm, Whatman GF/D, Cytiva, USA) was used and soaked with 100 µL of liquid electrolyte (LP57, Gotion Inc., USA) containing 1 mol LiPF_6_ and 2 vol.% vinylene carbonate. The cells were cycled at a C‐rate of 0.02 C to ideally address 100% of the active material. To determine the active mass of the SSB cells, a customized C‐rate procedure was used, and then the active mass was determined using a Python tool (Figure S12b, Supporting Information). A detailed description of the C‐rate procedure and the evaluation method can be found in Kissel et al.^[^ ^31^ ^]^

To determine partial conductivity values of the cathode composites, both ionic and electron blocking cells were assembled. For electron blocking conditions, 80 mg of the prepared composites were layered directly between two steel stamps. For ion blocking conditions, 80 mg of the composite powder was layered between 80 mg of LPSCl with In–Li‐alloy on both sides to form the following cell stack In/InLi|LPSCl|NCM: LPSCl|LPSCl|In/InLi. The argyrodite LPSCl was employed on both sides due to its good interfacial stability toward the In/InLi and to ensure no transport limitations in the electron blocking layer.^[^ ^83^ ^]^ The stack was carefully assembled under Ar‐atmosphere (*p*(H_2_O)/*p* < 0.5 ppm and *p*(O_2_)/*p *< 0.1 ppm) by hand‐pressing each layer before adding the next. Before the indium and lithium foils were added, a densification step using uniaxial pressure of 3 t (≈380 MPa) for 3 min was carried out to ensure good contact between the layers and the electrodes. A waiting time of 6 h was added after the cells were put into an aluminium frame with an applied pressure of 50 MPa. To evaluate the effective conductivity, a PEIS measurement of the symmetric cell stack was performed using an AC excitation voltage amplitude of 10 mV (*V*_rms_ = 7.07 mV) and recorded in a frequency range of 7 MHz to 10 mHz. Afterward, constant potentials of 1, 2, 5, 10, 15, 20, 25, 30, and 50 mV were applied for 1 h each. Equilibrium current values were extracted for all potentials and, assuming it can be solely attributed to ionic transport, fitted from *I*–*U* plots based on Ohm's law. Subtracting the resistance contributed to the LPSCl, and considering the sample geometry, the respective ionic conductivities are obtained.

### Dry Composite Film Production

For up‐scaled processing, the pre‐milled composites consisting of 70:30 NCM:LPSCl from the planetary ball mill were mixed with 3 wt.% of PTFE binder (Daikin Industries, Ltd.). The PTFE binder was added to the pre‐milled composite, and the whole mixture was stressed in a high‐intensity ring‐shear mixer (Hosokawa Alpine AG) for 5 min at 5000 rpm to create sufficient pre‐fibrillation of the PTFE. Subsequently, the mixture was calendered using a two‐roll calender (Saueressig GK300L, Matthews Europe GmbH). The material was compacted and sheared under force control with a roller force of 8 kN, a roller circumferential speed of 0.5 m min^−1^, a roller temperature of 100 °C, and a differential speed between the rolls, where the second roll operated at 60% of the first roll's speed (0.3 m min^−1^). After this, the formed free‐standing film was again calendered with a roller differential speed of 150%, meaning the second roll now rotated at 0.75 m min^−1^. Next, the film was applied to a C‐coated aluminum current collector (En'safe 91, Armor Battery Films) with both rollers at a circumferential speed of 0.1 m min^−1^. PTFE fibrillation and calendering took place in a dry room at 20 °C and a relative humidity of < 0.1% (dew point of the supply air *T*_D_ = − 60 °C).

### Raman Measurements

Raman spectra were recorded using an alpha300 R Raman imaging microscope (WITec GmbH) with an excitation wavelength of 532 nm and a laser power of 1 mW. A 600 g mm^−1^ grating (BLZ = 500 nm) was used, and the integration time was set to 2 s for 60 data points. In total, five spectra were recorded for each sample. To prevent air exposure, the sample was prepared in an argon‐filled glovebox by placing it on a glass slide, sealing it with a ring of silicone paste, and covering it with a glass cover slip to ensure an airtight enclosure.

## Conflict of Interest

The authors declare no conflict of interest.

## Supporting information



Supporting Information

## Data Availability

The data that support the findings of this study are available from the corresponding author upon reasonable request.
