# Supplementary material for: Tailoring Composite Microstructure Through Milling for Dry‐Processed Sulfide‐Based Solid‐State Battery Cathodes
Source: Small. 2025 Sep 5;21(41):e07279. doi: 10.1002/smll.202507279 (PMC12530022; doi:10.1002/smll.202507279)
Supplement: Supplementary file 1 — Supporting Information [file SMLL-21-e07279-s001.docx]

Supporting Information

Tailoring Composite Microstructure through Milling for Dry-Processed Sulfide-Based Solid-State Battery Cathodes

Finn Frankenberg^a*^, Carina A. Heck^a^, Maximilian Kissel^b^, Martin A. Lange^d^, Vasiliki Faka^c^, Alexander Diener^a^, Philipp Haase^a^, Peter Michalowski^a^, Wolfgang G. Zeier^c,d^, Jürgen Janek^b^, and Arno Kwade^a^

^a^Technische Universität Braunschweig, Institute for Particle Technology, Volkmaroder Straße 5,

Braunschweig, 38104, Germany, Lower Saxony

^b^Justus-Liebig-Universität Gießen, Institute of Physical Chemistry & Center for Materials Research, Heinrich-Buff-Ring 17, Gießen, 35392, Germany, Hesse

^c^Universität Münster, Institute of Inorganic and Analytical Chemistry, Corrensstraße 28-30, Münster, 48149, Germany, North Rhine-Westphalia

^d^Forschungszentrum Jülich GmbH, Institute of Energy Materials and Devices (IMD), IMD 4: Helmholtz-Institut Münster Ionics in Energy Storage, Münster, 48149, Germany, North Rhine-Westphalia

Corresponding author: Finn Frankenberg, [finn.frankenberg@tu-braunschweig.de](mailto:finn.frankenberg@tu-braunschweig.de)

|  |
| --- |
|  |

| 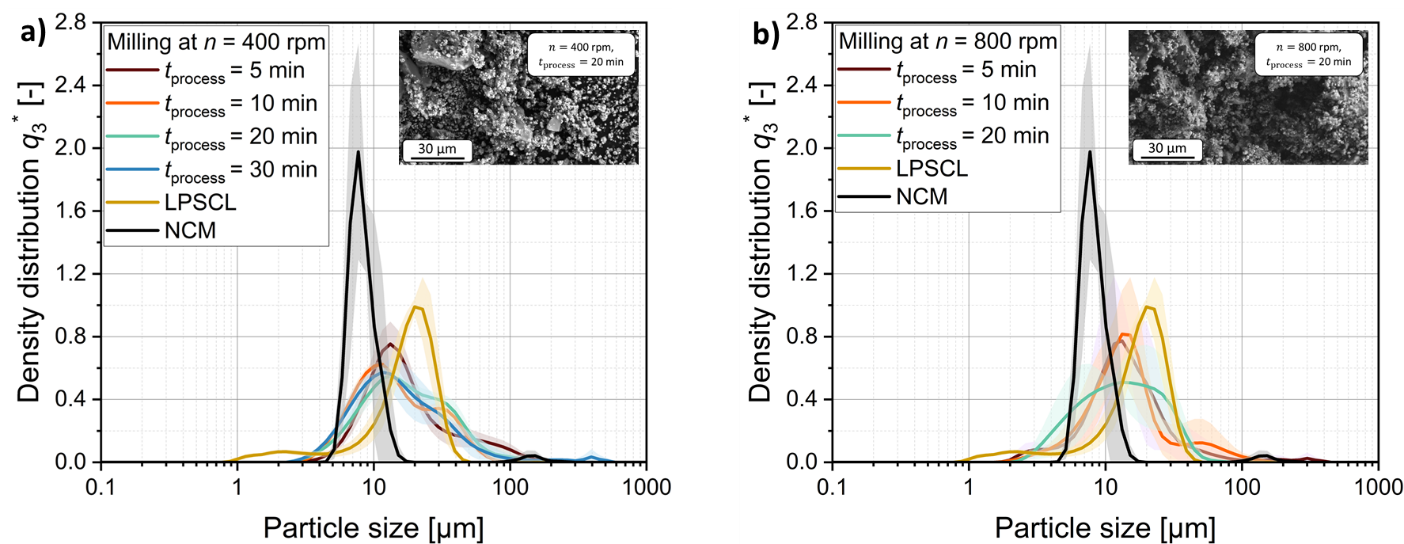 |
| --- |
| **Figure S1**: Volume-based particle size distributions and SEM images of the NCM – LPSCl composites processed at different rotational speeds. a) Processing at 400 rpm for 5 minutes to 30 minutes. b) Processing at 800 rpm for 5 minutes to 20 minutes. |
|  |
| 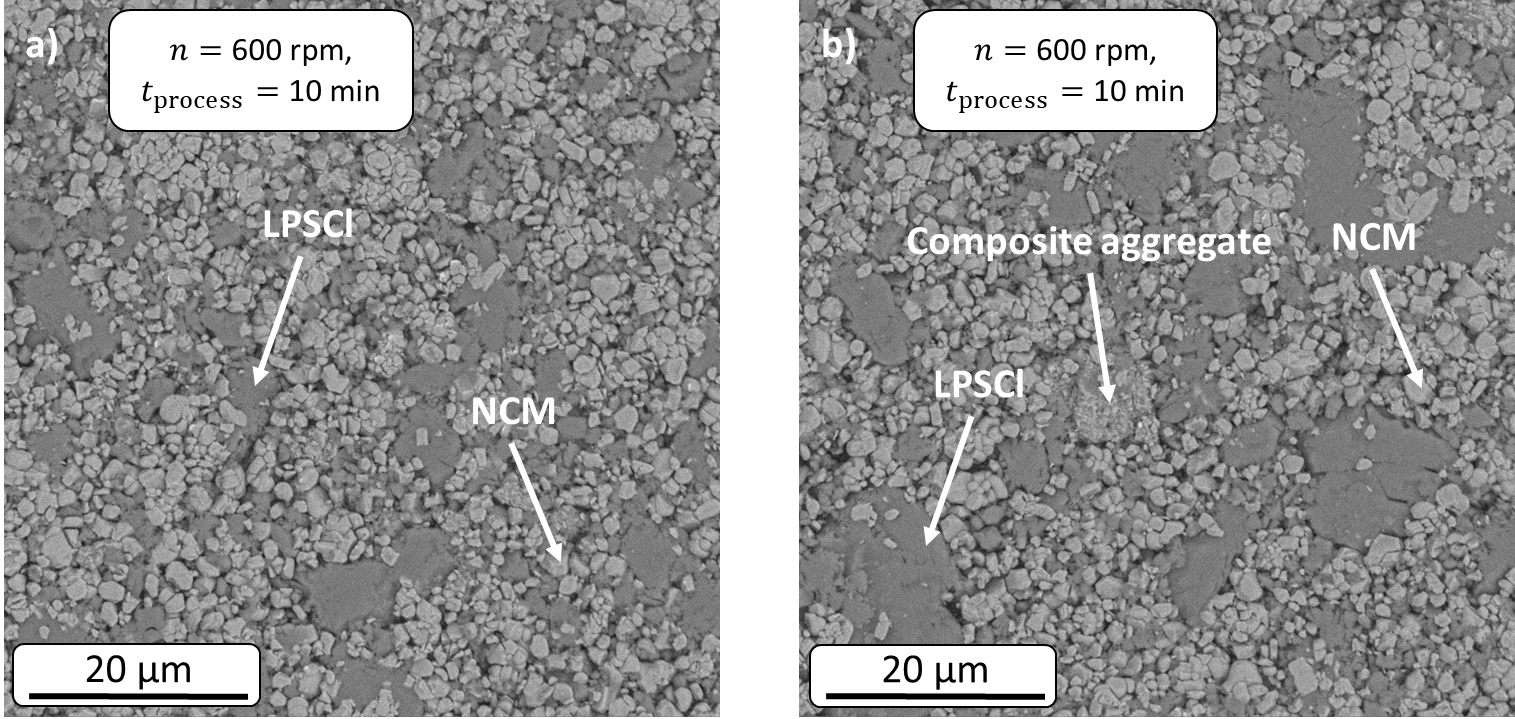 |
| **Figure S2**: a) and b) Top-view images of the pressed cathode composites produced at 600 rpm for 10 minutes. |

| **Table S1:** Mean stress energy and stress frequency of the ball milling process simulated for a filling ratio of 0.5. | | | | | | |
| --- | --- | --- | --- | --- | --- | --- |
|  | $\boldsymbol{\varphi=}$ **0.5** | | | | | |
|  | **5 mm** | | **3 mm** | | **1.75 mm** | |
| $n_{\mathrm{MC}}$ | $\bar{SE}$ [J] | $SF$ [s^-1^] | $\bar{SE}$ [J] | $SF$ [s^-1^] | $\bar{SE}$ [J] | $SF$ [s^-1^] |
| 200 | 5.38478E-07 | 1232846 | 9.06932E-08 | 10135490 | 1.18E-08 | 89957880 |
| 400 | 3.25615E-06 | 1471680 | 4.63296E-07 | 10923396 | 6.24E-08 | 93135940 |
| 600 | 9.876E-06 | 1671156 | 1.29752E-06 | 12790754 | 1.67E-07 | 110175704 |
| 800 | 2.05545E-05 | 1990424 | 2.96358E-06 | 14166094 | 3.64E-07 | 124078208 |
| 1000 | 3.7592E-05 | 1837780 | 4.88483E-06 | 15534152 | 6.74E-07 | 139648252 |

| **Table S2:** Mean stress energy and stress frequency of the ball milling process simulated for a filling ratio of 0.3. | | | | | | |
| --- | --- | --- | --- | --- | --- | --- |
|  | $\boldsymbol{\varphi=}$ **0.3** | | | | | |
|  | **5 mm** | | **3 mm** | | **1.75 mm** | |
| $n_{\mathrm{MC}}$ | $\bar{SE}$ [J] | $SF$ [s^-1^] | $\bar{SE}$ [J] | $SF$ [s^-1^] | $\bar{SE}$ [J] | $SF$ [s^-1^] |
| 200 | 1.5888E-06 | 696874 | 2.0788E-07 | 6010610 | 2.16E-08 | 61629742 |
| 400 | 8.604E-06 | 1141350 | 1.1759E-06 | 9315414 | 1.26E-07 | 92381462 |
| 600 | 2.2192E-05 | 1517998 | 3.1325E-06 | 11889102 | 3.45E-07 | 114602902 |
| 800 | 4.3518E-05 | 1837064 | 6.2399E-06 | 14160526 | 7.03E-07 | 133245338 |
| 1000 | 7.2494E-05 | 2168506 | 1.063E-05 | 16291870 | 1.22E-06 | 150205456 |

| **Table S3:** Mean stress energy and stress frequency of the ball milling process simulated for a filling ratio of 0.1. | | | | | | |
| --- | --- | --- | --- | --- | --- | --- |
|  | $\boldsymbol{\varphi=}$ **0.1** | | | | | |
|  | **5 mm** | | **3 mm** | | **1.75 mm** | |
| $n_{\mathrm{MC}}$ | $\bar{SE}$ [J] | $SF$ [s^-1^] | $\bar{SE}$ [J] | $SF$ [s^-1^] | $\bar{SE}$ [J] | $SF$ [s^-1^] |
| 200 | 1.169E-06 | 317832 | 2.4968E-07 | 1815560 | 3.02E-08 | 16213938 |
| 400 | 5.2661E-06 | 677714 | 1.1373E-06 | 3341530 | 1.67E-07 | 23776736 |
| 600 | 1.4424E-05 | 862280 | 2.7274E-06 | 4695922 | 4.49E-07 | 30115112 |
| 800 | 2.749E-05 | 1082420 | 5.689E-06 | 5395322 | 8.96E-07 | 35883770 |
| 1000 | 4.3617E-05 | 1323844 | 9.3094E-06 | 6439074 | 1.54E-06 | 40930020 |

| 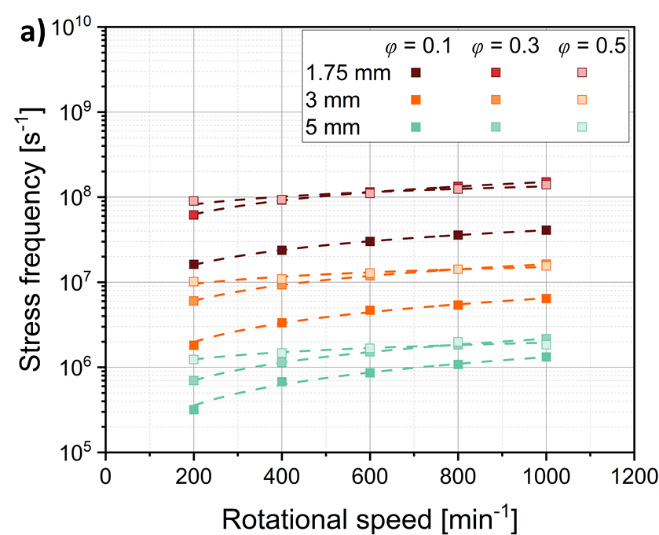 | 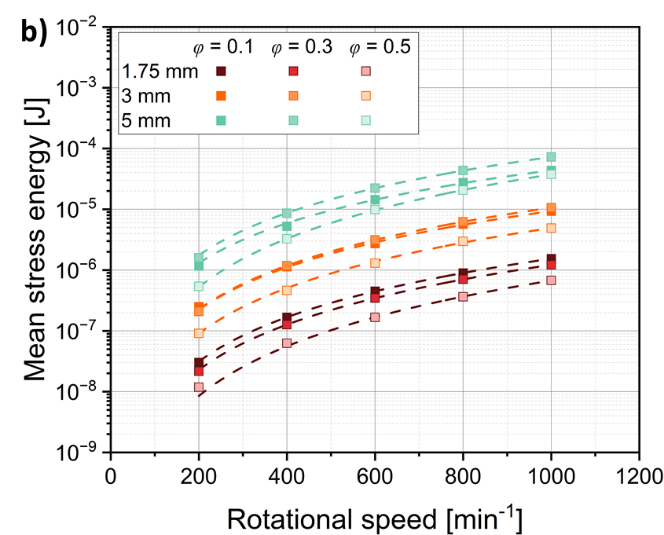 |
| --- | --- |
| **Figure S3**: a) Influence of the rotational speed of the milling chamber on the stress frequency $SF$. b) Influence of the rotational speed of the milling chamber on the stress energy $\bar{SE}$. | |

| 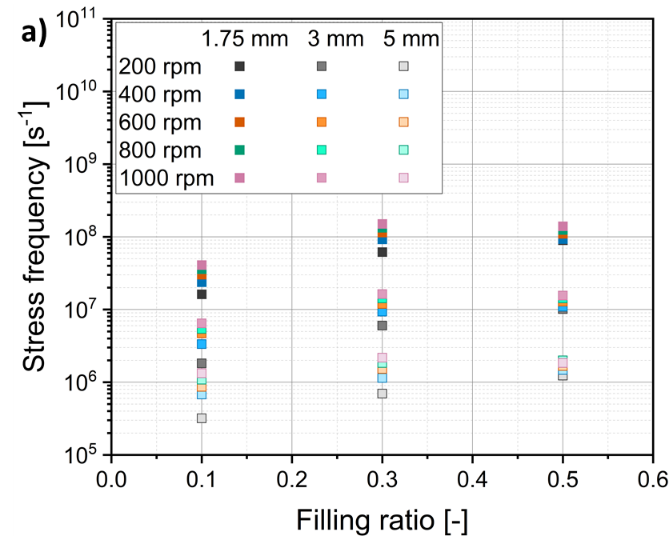 | 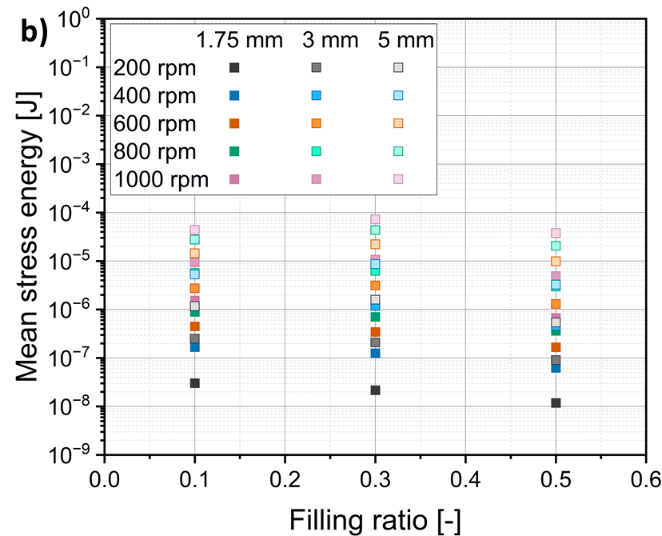 |
| --- | --- |
| **Figure S4**: a) Influence of the milling bead filling ratio on the stress frequency $SF$. b) Influence of the milling beads filling ratio on the stress energy $\bar{SE}$. | |

| **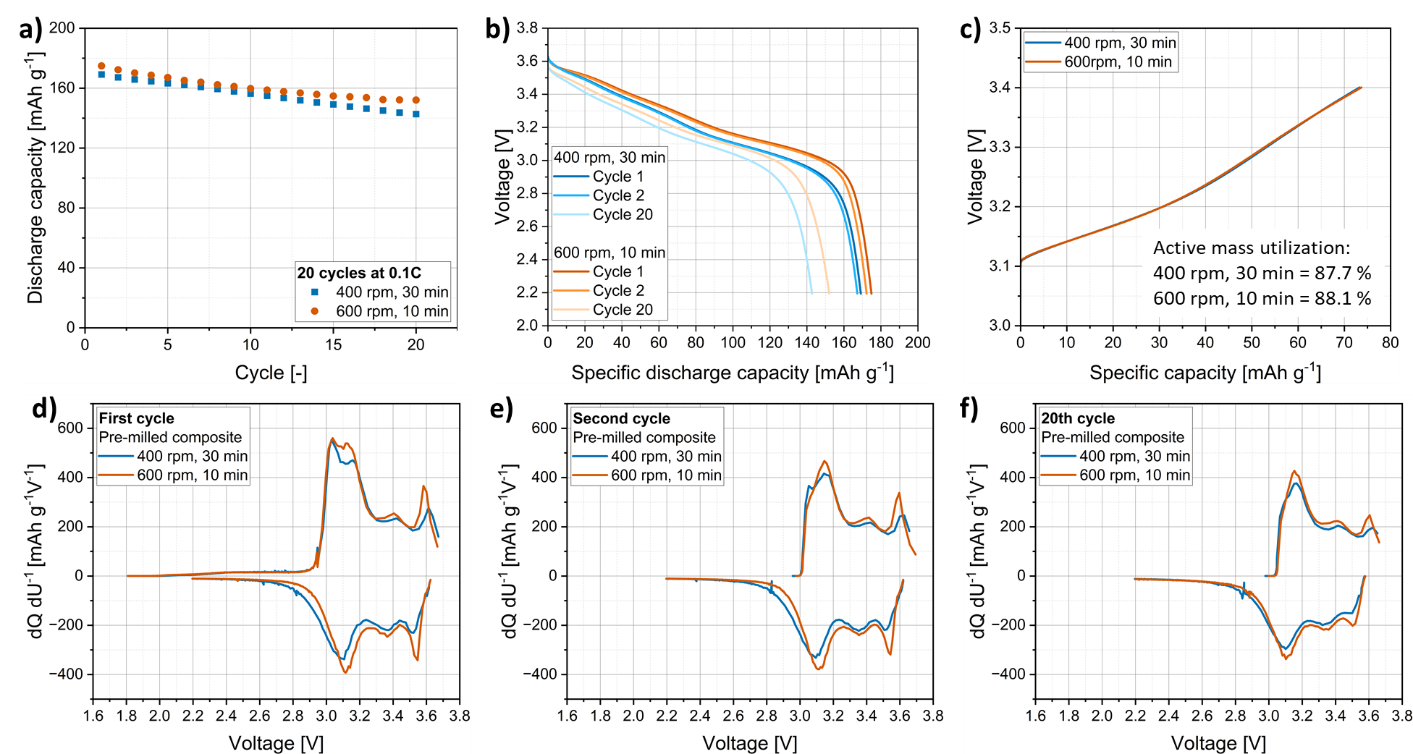** |
| --- |
| **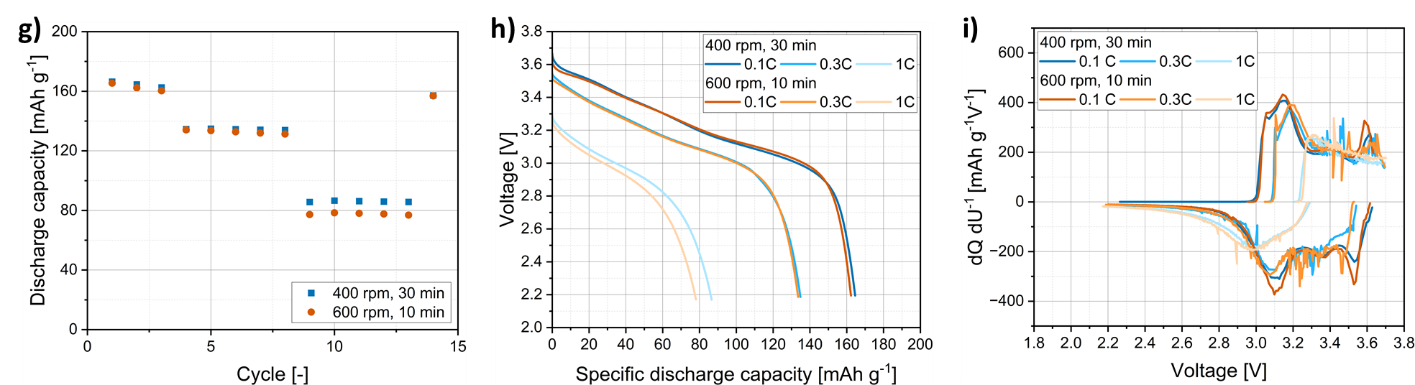** |
| **Figure S5**: Detailed comparison of composites produced at 600 rpm and 10 min and 400 rpm and 30 min milling time. All electrochemical analyses show a high degree of consistency within the standard deviation, which is due in particular to the cell assembly. |

| **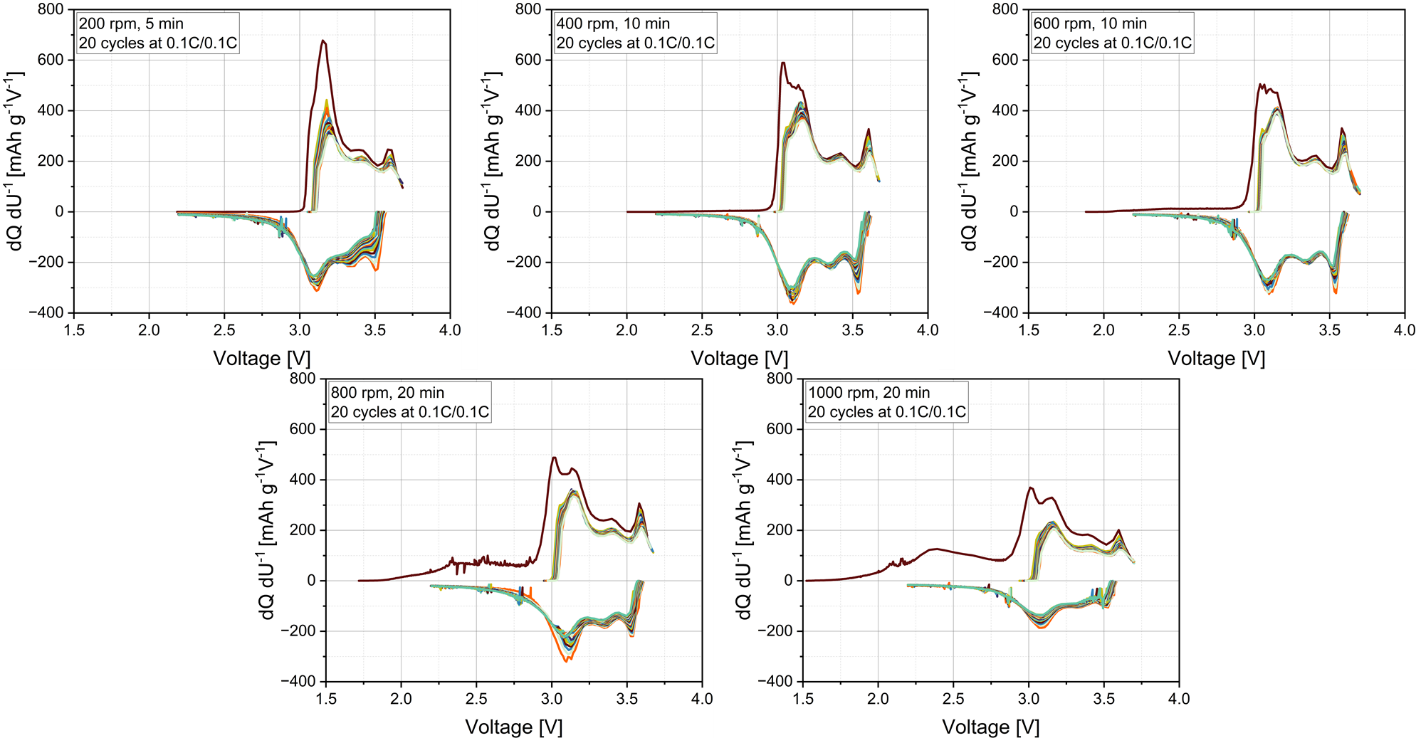** |
| --- |
| **Figure S6**: dQ dU^-1^ diagrams of different composites cycled for 20 cycles at 0.1C/0.1C. |

| 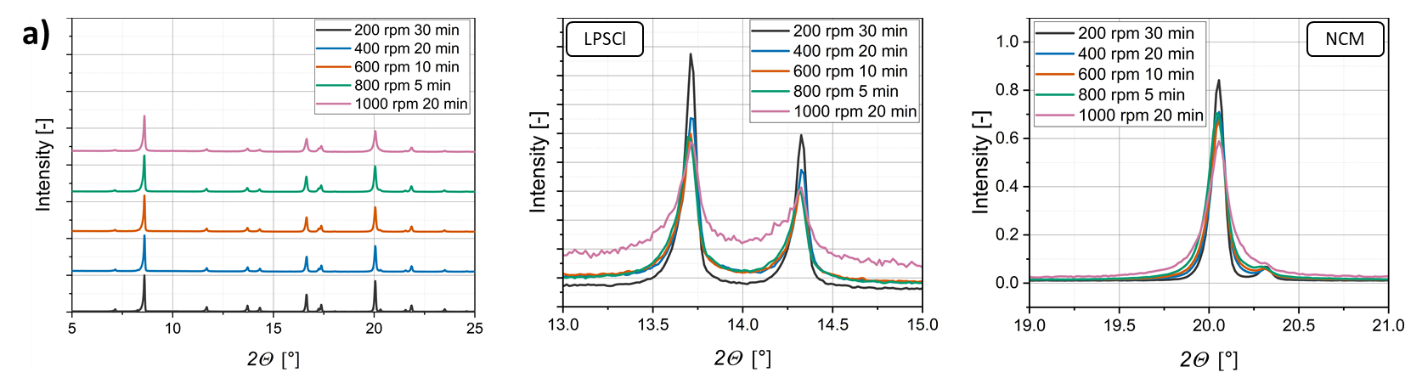 | |
| --- | --- |
|  |  |
| **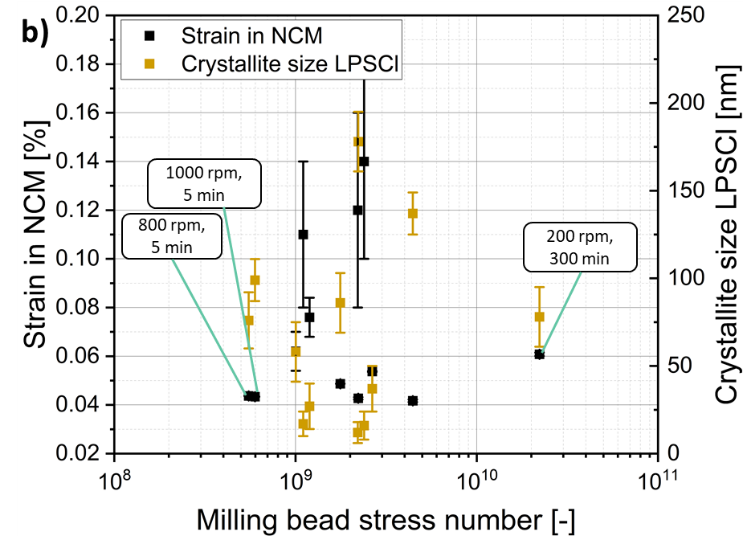** | |
| **Figure S7**: a) XRD analysis of cathode composites produced at different rotational speeds for different milling times. b) Influence of stress number $SN$ on the NCM strain and LPSCl crystallite size. | |

| 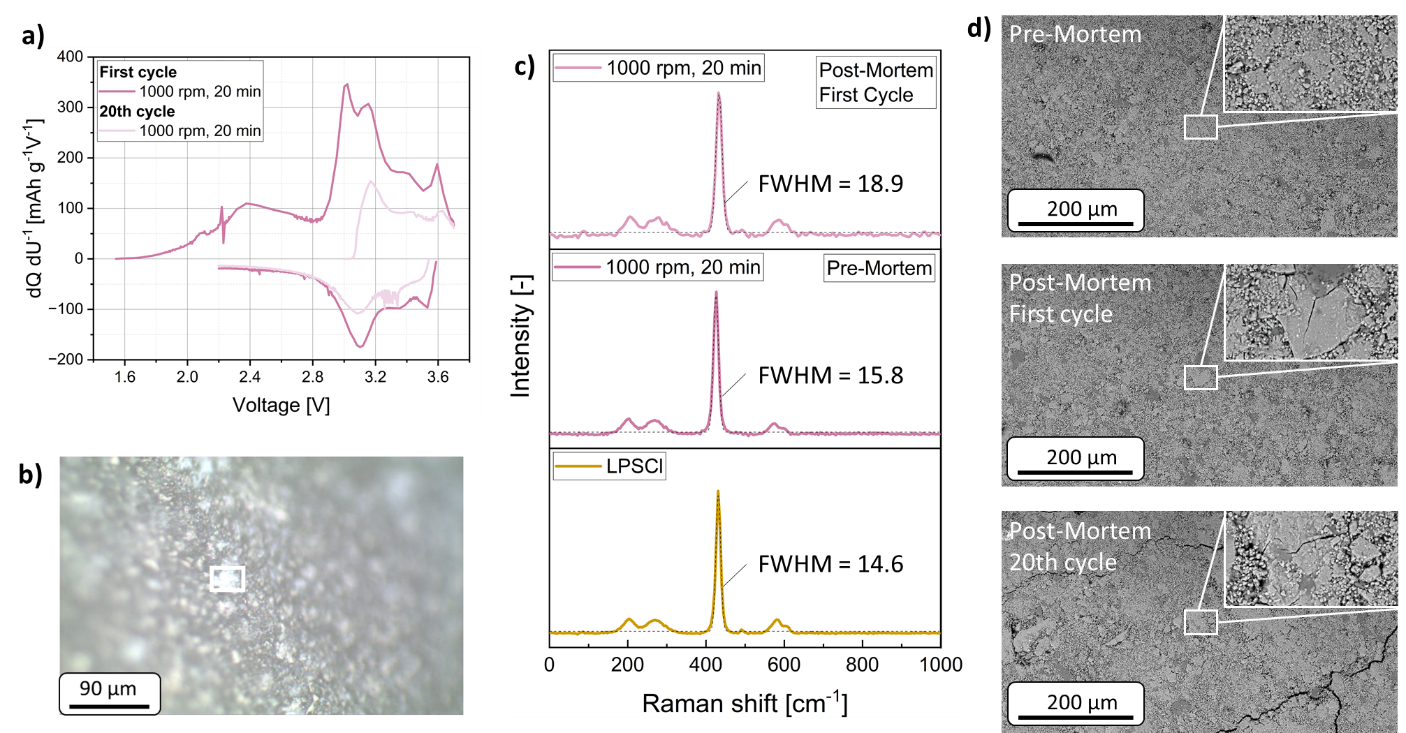 |
| --- |
| **Figure S8**: a) dQ dU^-1^ plots of the highly stressed composite (1000 rpm, 20 min) after 1 and 20 cycles. b) Optical image showing a Raman measurement spot. c) Raman spectra of pure LPSCl, LPSCl within the highly stressed composite (pre-mortem), and post-mortem (after 1 cycle). d) SEM images of the composite surface: pre-mortem, post-mortem after 1 cycle, and post-mortem after 20 cycles. |

| 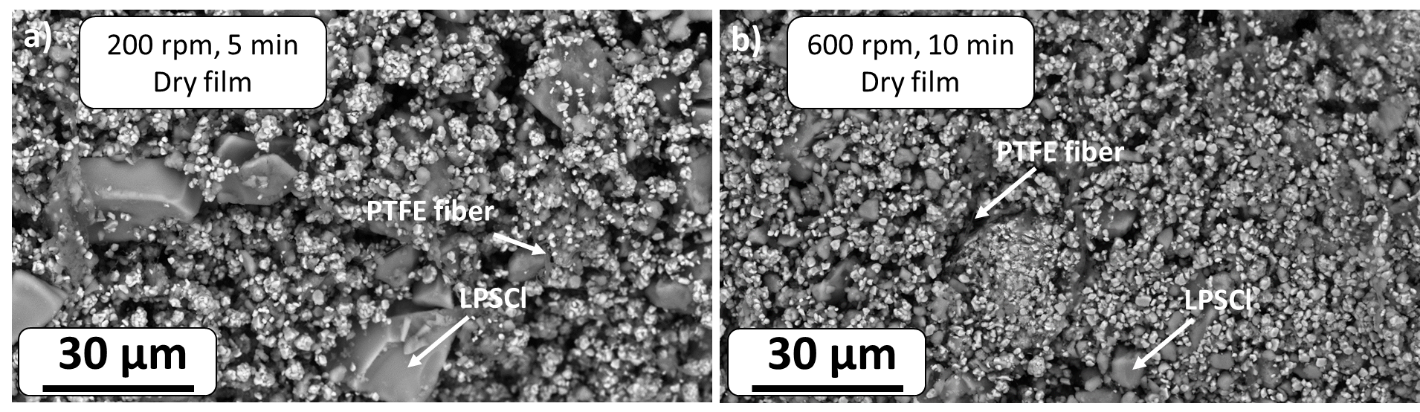 |
| --- |
| **Figure S9**: SEM cross-section of the dry produced films. a) Cross section from the film that was produced with the composite which was milled at 200 rpm for 5 minutes. b) Cross section from the film that was produced with the composite which was milled at 600 rpm for 10 minutes. |

| 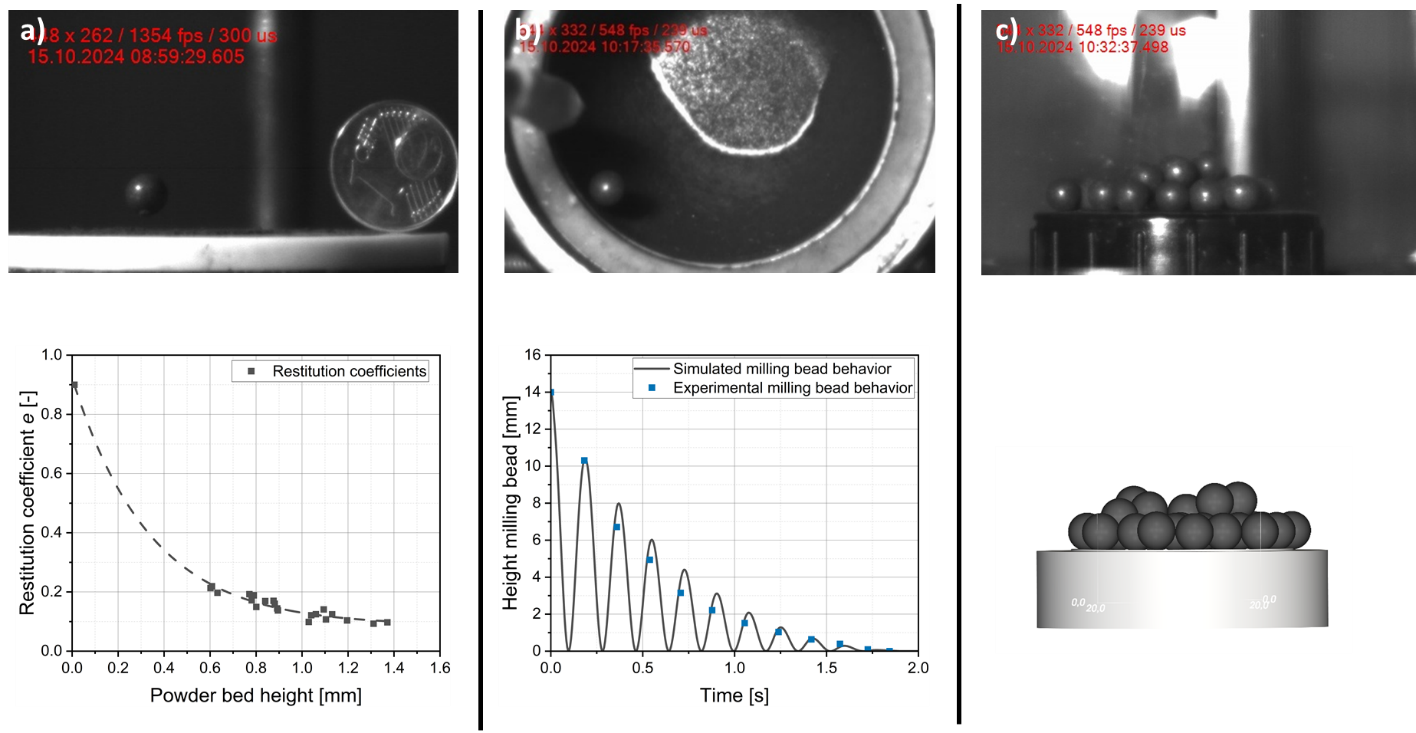 |
| --- |
| **Figure S10**: Calibration experiments for milling beads. a) Drop-down test for the determination of the restitution coefficient. b) Rolling friction in the milling chamber: Experiment and simulation. c) Static angle of repose determination. |

| 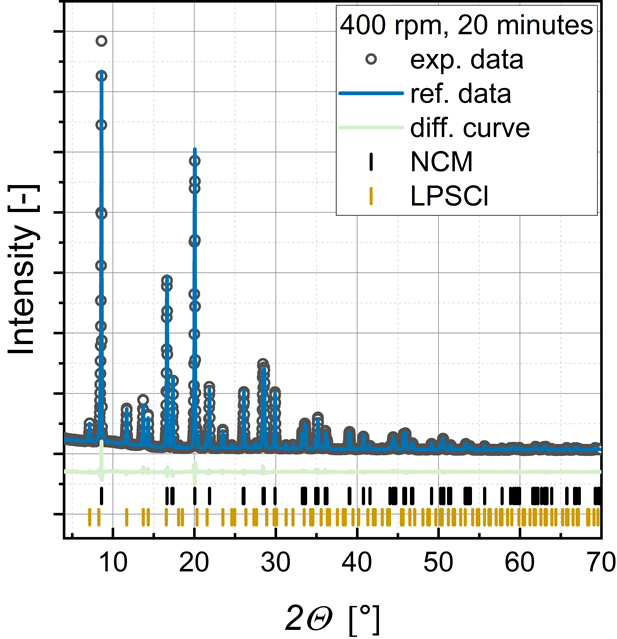 |
| --- |
| **Figure S11**: Example plot for an exemplary Rietveld Refinement for PXRD data of the sample milled at 400 rpm for 20 minutes. |

| **Table S4:** Crystallographic data of NCM and LPSCl as used to refine Mo-X-Ray diffraction data, indicating applied restraints as variables. Fixed values for occupancies for both materials were obtained by refining against diffraction data of the pristine materials or, in case of the lithium site occupancies in LPSCl, taken from literature. Statistical standard deviation for each refined parameter is shown in parentheses.   \| NCM \| \| \| \| \| *λ*_Molybdenum_ = 0.7093 Å \| \| \| --- \| --- \| --- \| --- \| --- \| --- \| --- \| \| Atom \| Wyckoff site \| *x* \| *y* \| *z* \| Occupancy \| *B*_eq_ \| \| O \| 6*c* \| 0 \| 0 \| *zO* \| 1 \| bisoO \| \| Li1 \| 3*b* \| 0 \| 0 \| 0.5 \| 0.9892(9) \| bisoLi \| \| Ni1 \| 3*b* \| 0 \| 0 \| 0.5 \| 0.0118(9) \| bisoLi \| \| Ni2 \| 3*a* \| 0 \| 0 \| 0 \| 0.924(2) \| bisoNi \| \| Li2 \| 3*a* \| 0 \| 0 \| 0 \| 0.073(2) \| bisoNi \| \| Li_6_PS_5_Cl \| \| \| \| \| \| \| \| S1 \| 4*d* \| 0.25 \| 0.25 \| 0.75 \| 0.86(4) \| bisoS \| \| Cl1 \| 4*d* \| 0.25 \| 0.25 \| 0.75 \| 0.14(4) \| bisoS \| \| S2 \| 4*a* \| 0 \| 0 \| 1 \| 0.14(4) \| bisoS \| \| Cl2 \| 4*a* \| 0 \| 0 \| 1 \| 0.86(4) \| bisoS \| \| S3 \| 16*e* \| *xS* \| -*xS* \| 0.5 + *xS* \| 1 \| bisoS \| \| P \| 4*b* \| 0 \| 0 \| 0.5 \| 1 \| bisoP \| \| Li1 \| 48*h* \| 0.260 \| 0.4515 \| 0.9515 \| 0.1 \| 2.5 \| \| Li2 \| 48*h* \| 0.3179 \| 0.0233 \| 0.6821 \| 0.395 \| 4.2 \|  \| **Table S5:** Crystallographic data of NCM and LPSCl as obtained from refinements against Mo-X-Ray diffraction data for composites milled at different speeds and times. Listed are all refined values, as indicated in Table S4. Statistical standard deviation for each refined parameter is shown in parentheses. \| \| \| \| \| \| \| \| \| \| \| --- \| --- \| --- \| --- \| --- \| --- \| --- \| --- \| --- \| --- \| \| NCM  *λ*_Molybdenum_ = 0.7093 Å \| \| \| \| \| \| \| \| \| \| \| Milling speed [min^-1^] \| Milling time [min] \| *a* \| *c* \| \| *zO* \| bisoO \| bisoLi \| \| bisoNi \| \| 200 \| 30 \| 2.87211(2) \| 14.1899(1) \| \| 0.25835(9) \| 0.90(3) \| 1.1(1) \| \| 0.172(8) \| \| 200 \| 60 \| 2.87201(2) \| 14.1886(1) \| \| 0.25844(8) \| 0.88(3) \| 1.1(1) \| \| 0.188(7) \| \| 200 \| 300 \| 2.87218(2) \| 14.1882(2) \| \| 0.25840(9) \| 0.80(3) \| 1.1(1) \| \| 0.135(8) \| \| 400 \| 20 \| 2.87222(2) \| 14.1889(2) \| \| 0.25841(9) \| 0.78(4) \| 1.0(1) \| \| 0.164(8) \| \| 400 \| 30 \| 2.87207(4) \| 14.1884(3) \| \| 0.2584(1) \| 0.71(6) \| 1.3(2) \| \| 0.18(1) \| \| 600 \| 10 \| 2.87203(3) \| 14.1879(2) \| \| 0.25847(9) \| 0.76(4) \| 1.1(1) \| \| 0.134(9) \| \| 800 \| 5 \| 2.87220(2) \| 14.1892(2) \| \| 0.25834(7) \| 0.80(3) \| 1.4(1) \| \| 0.111(7) \| \| 800 \| 10 \| 2.87217(3) \| 14.1882(3) \| \| 0.2581(1) \| 0.66(4) \| 1.5(1) \| \| 0.12(1) \| \| 800 \| 20 \| 2.87511(7) \| 14.1937(7) \| \| 0.2587(2) \| 0.68(6) \| 1.3(2) \| \| 0.12(2) \| \| 1000 \| 5 \| 2.87228(2) \| 14.1893(2) \| \| 0.2585(1) \| 0.82(4) \| 1.1(1) \| \| 0.160(9) \| \| 1000 \| 10 \| 2.87235(6) \| 14.1886(6) \| \| 0.2583(2) \| 0.73(8) \| 1.6(2) \| \| 0.11(3) \| \| 1000 \| 20 \| 2.87192(7) \| 14.1853(7) \| \| 0.2577(2) \| 0.87(7) \| 1.0(2) \| \| 0.18(2) \| \| Li_6_PS_5_Cl \| \| \| \| \| \| \| \| \| \| \| Milling speed [min^-1^] \| Milling time [min] \| *a* \| \| *xS* \| \| beqS \| \| beqP \| \| \| 200 \| 30 \| 9.8488(1) \| \| 0.1192(2) \| \| 2.94(7) \| \| 2.3(1) \| \| \| 200 \| 60 \| 9.8507(1) \| \| 0.1993(2) \| \| 2.95(7) \| \| 2.2(1) \| \| \| 200 \| 300 \| 9.8513(2) \| \| 0.1199(3) \| \| 2.8(1) \| \| 2.1(2) \| \| \| 400 \| 20 \| 9.8487(2) \| \| 0.1200(3) \| \| 3.0(1) \| \| 2.4(2) \| \| \| 400 \| 30 \| 9.8482(3) \| \| 0.1200(6) \| \| 3.8(2) \| \| 3.8(4) \| \| \| 600 \| 10 \| 9.8509(3) \| \| 0.1198(4) \| \| 3.0(1) \| \| 2.3(2) \| \| \| 800 \| 5 \| 9.8509(1) \| \| 0.1197(2) \| \| 2.88(7) \| \| 2.2(1) \| \| \| 800 \| 10 \| 9.8519(3) \| \| 0.1197(4) \| \| 3.6(1) \| \| 3.3(3) \| \| \| 800 \| 20 \| 9.8523(9) \| \| 0.120(1) \| \| 3.9(4) \| \| 3.8(9) \| \| \| 1000 \| 5 \| 9.8494(2) \| \| 0.1200(3) \| \| 3.0(1) \| \| 2.2(2) \| \| \| 1000 \| 10 \| 9.8534(7) \| \| 0.121(1) \| \| 4.2(3) \| \| 3.9(5) \| \| \| 1000 \| 20 \| 9.8578(9) \| \| 0.1199(9) \| \| 3.7(4) \| \| 3.5(6) \| \|  \| **Table S6:** Refined $e_{0}$-microstrain values and integral breadth ($L_{\mathrm{Vol}}$) of NCM and LPSCl as obtained from refinements against Mo-X-Ray diffraction data for composites milled at different speeds and times according to Balzar et. al.^[1]^ No values for the integral breadth of NCM are listed since no broadening due to reduced crystal domain size could be observed. In addition, the quality parameters of the refinements $R_{\mathrm{wp}}$ and GoF are listed. Statistical standard deviation for each refined parameter is shown in parentheses.  [1] D. Balzar, N. Audebrand, M. R. Daymond, A. Fitch, A. Hewat, J. I. Langford, A. Le Bail, D. Louër, O. Masson, C. N. McCowan, N. C. Popa, P. W. Stephens, B. H. Toby, *J Appl Crystallogr* **2004**, *37*, 911. \| \| \| \| \| \| \| \| --- \| --- \| --- \| --- \| --- \| --- \| --- \| \| *λ*_Molybdenum_ = 0.7093 Å \| \| \| \| \| \| \| \| Milling speed [min^-1^] \| Milling time [min] \| $e_{0}$(NCM) [%] \| $e_{0}$(LPSCl) [%] \| $L_{\mathrm{Vol}}$(LPSCl) [nm] \| $R_{\mathrm{wp}}$ \| GoF \| \| 200 \| 30 \| 0.0427(5) \| 0.026(9) \| 178(17) \| 4.8 \| 2.2 \| \| 200 \| 60 \| 0.0417(5) \| 0.03(9) \| 137(12) \| 4.0 \| 2.1 \| \| 200 \| 300 \| 0.0608(7) \| 0.05(1) \| 78(17) \| 4.5 \| 2.6 \| \| 400 \| 20 \| 0.0487(1) \| 0.05(1) \| 86(17) \| 4.5 \| 2.5 \| \| 400 \| 30 \| 0.0537(2) \| 0.0004(9) \| 37(13) \| 7.3 \| 1.1 \| \| 600 \| 10 \| 0.062(8) \| 0.05(2) \| 58(17) \| 4.6 \| 2.3 \| \| 800 \| 5 \| 0.0434(1) \| 0.04(1) \| 99(12) \| 4.1 \| 2.2 \| \| 800 \| 10 \| 0.076(8) \| 0.004(9) \| 27(13) \| 5.2 \| 2.6 \| \| 800 \| 20 \| 0.14(4) \| 0.0004(9) \| 16(8) \| 6.0 \| 3.1 \| \| 1000 \| 5 \| 0.0437(1) \| 0.04(2) \| 76(16) \| 4.9 \| 3.6 \| \| 1000 \| 10 \| 0.11(3) \| 0.0004(9) \| 17(7) \| 6.6 \| 1.4 \| \| 1000 \| 20 \| 0.12(4) \| 0.0005(8) \| 12(6) \| 6.6 \| 3.3 \| |
| --- | --- | --- | --- | --- | --- | --- | --- | --- | --- | --- | --- | --- | --- | --- | --- | --- | --- | --- | --- | --- | --- | --- | --- | --- | --- | --- | --- | --- | --- | --- | --- | --- | --- | --- | --- | --- | --- | --- | --- | --- | --- | --- | --- | --- | --- | --- | --- | --- | --- | --- | --- | --- | --- | --- | --- | --- | --- | --- | --- | --- | --- | --- | --- | --- | --- | --- | --- | --- | --- | --- | --- | --- | --- | --- | --- | --- | --- | --- | --- | --- | --- | --- | --- | --- | --- | --- | --- | --- | --- | --- | --- | --- | --- | --- | --- | --- | --- | --- | --- | --- | --- | --- | --- | --- | --- | --- | --- | --- | --- | --- | --- | --- | --- | --- | --- | --- | --- | --- | --- | --- | --- | --- | --- | --- | --- | --- | --- | --- | --- | --- | --- | --- | --- | --- | --- | --- | --- | --- | --- | --- | --- | --- | --- | --- | --- | --- | --- | --- | --- | --- | --- | --- | --- | --- | --- | --- | --- | --- | --- | --- | --- | --- | --- | --- | --- | --- | --- | --- | --- | --- | --- | --- | --- | --- | --- | --- | --- | --- | --- | --- | --- | --- | --- | --- | --- | --- | --- | --- | --- | --- | --- | --- | --- | --- | --- | --- | --- | --- | --- | --- | --- | --- | --- | --- | --- | --- | --- | --- | --- | --- | --- | --- | --- | --- | --- | --- | --- | --- | --- | --- | --- | --- | --- | --- | --- | --- | --- | --- | --- | --- | --- | --- | --- | --- | --- | --- | --- | --- | --- | --- | --- | --- | --- | --- | --- | --- | --- | --- | --- | --- | --- | --- | --- | --- | --- | --- | --- | --- | --- | --- | --- | --- | --- | --- | --- | --- | --- | --- | --- | --- | --- | --- | --- | --- | --- | --- | --- | --- | --- | --- | --- | --- | --- | --- | --- | --- | --- | --- | --- | --- | --- | --- | --- | --- | --- | --- | --- | --- | --- | --- | --- | --- | --- | --- | --- | --- | --- | --- | --- | --- | --- | --- | --- | --- | --- | --- | --- | --- | --- | --- | --- | --- | --- | --- | --- | --- | --- | --- | --- | --- | --- | --- | --- | --- | --- | --- | --- | --- | --- | --- | --- | --- | --- | --- | --- | --- | --- | --- | --- | --- | --- | --- | --- | --- | --- | --- | --- | --- | --- | --- | --- | --- | --- | --- | --- | --- | --- | --- | --- | --- | --- | --- | --- | --- | --- | --- | --- | --- | --- | --- | --- | --- | --- | --- | --- | --- | --- | --- | --- | --- | --- | --- | --- | --- | --- | --- | --- | --- | --- | --- | --- | --- | --- | --- | --- | --- | --- | --- | --- | --- | --- | --- | --- | --- | --- | --- | --- | --- | --- | --- | --- | --- | --- | --- | --- | --- | --- | --- | --- | --- | --- | --- | --- | --- | --- | --- | --- | --- | --- | --- | --- | --- | --- | --- | --- | --- | --- | --- | --- | --- | --- | --- | --- | --- | --- | --- | --- | --- | --- | --- | --- | --- | --- | --- | --- | --- | --- | --- | --- | --- | --- | --- | --- | --- | --- | --- | --- | --- | --- | --- | --- | --- | --- | --- | --- | --- | --- | --- | --- | --- | --- | --- | --- | --- | --- | --- | --- | --- | --- | --- | --- | --- | --- | --- | --- | --- | --- |

| **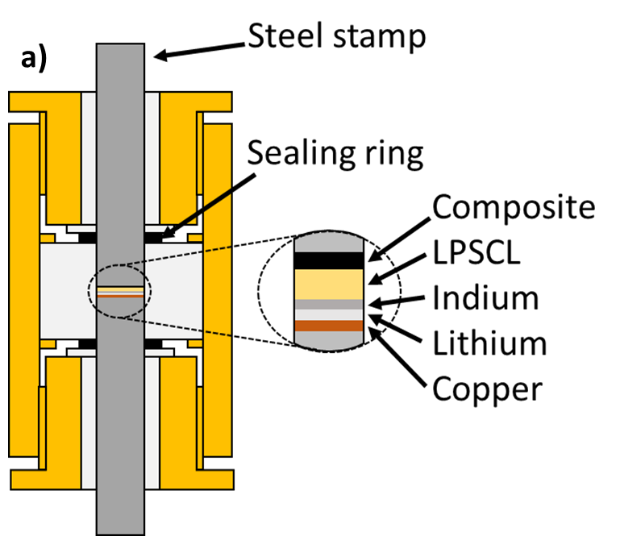** | **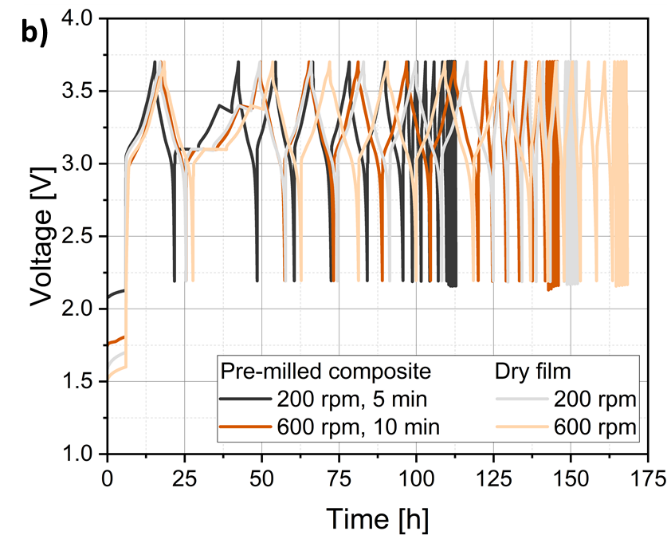** |
| --- | --- |
| **Figure S12**: a) Cell set-up for cyclization tests of powders and dry produced films. b) Voltage over time curves for the active mass determination of the pre-milled composites (200 rpm, 5 minutes & 600 rpm, 10 minutes) and the produced dry films from these composites. | |
